# Human cyclophilin 40 unravels neurotoxic amyloids

**DOI:** 10.1371/journal.pbio.2001336

**Published:** 2017-06-27

**Authors:** Jeremy D. Baker, Lindsey B. Shelton, Dali Zheng, Filippo Favretto, Bryce A. Nordhues, April Darling, Leia E. Sullivan, Zheying Sun, Parth K. Solanki, Mackenzie D. Martin, Amirthaa Suntharalingam, Jonathan J. Sabbagh, Stefan Becker, Eckhard Mandelkow, Vladimir N. Uversky, Markus Zweckstetter, Chad A. Dickey, John Koren, Laura J. Blair

**Affiliations:** 1 Department of Molecular Medicine and Byrd Alzheimer’s Institute, University of South Florida, Tampa, Florida, United States of America; 2 James A. Haley Veteran's Hospital, Tampa, Florida, United States of America; 3 German Center for Neurodegenerative Diseases (DZNE), Göttingen, Germany; 4 Department for NMR-based Structural Biology, Max Planck Institute for Biophysical Chemistry, Göttingen, Germany; 5 German Center for Neurodegenerative Diseases (DZNE), Bonn, Germany; 6 CAESAR Research Center, Bonn, Germany; 7 MPI for Metabolism Research, Hamburg, Germany; 8 Department of Neurology, University Medical Center Göttingen, Göttingen, Germany; University College London, United Kingdom of Great Britain and Northern Ireland

## Abstract

The accumulation of amyloidogenic proteins is a pathological hallmark of neurodegenerative disorders. The aberrant accumulation of the microtubule associating protein tau (MAPT, tau) into toxic oligomers and amyloid deposits is a primary pathology in tauopathies, the most common of which is Alzheimer’s disease (AD). Intrinsically disordered proteins, like tau, are enriched with proline residues that regulate both secondary structure and aggregation propensity. The orientation of proline residues is regulated by cis/trans peptidyl-prolyl isomerases (PPIases). Here we show that cyclophilin 40 (CyP40), a PPIase, dissolves tau amyloids in vitro. Additionally, CyP40 ameliorated silver-positive and oligomeric tau species in a mouse model of tau accumulation, preserving neuronal health and cognition. Nuclear magnetic resonance (NMR) revealed that CyP40 interacts with tau at sites rich in proline residues. CyP40 was also able to interact with and disaggregate other aggregating proteins that contain prolines. Moreover, CyP40 lacking PPIase activity prevented its capacity for disaggregation in vitro. Finally, we describe a unique structural property of CyP40 that may permit disaggregation to occur in an energy-independent manner. This study identifies a novel human protein disaggregase and, for the first time, demonstrates its capacity to dissolve intracellular amyloids.

## Author summary

Inside the cell, proteins need to be folded to be functional and active. Molecular chaperones are key enzymes that assist in folding proteins by stabilizing nascent polypeptide chains and by facilitating interactions that help stabilize a final structure. These chaperones also prevent the aggregation of newly formed proteins and can shunt misfolded proteins toward degradation pathways. In addition to interacting with newly synthesized proteins, chaperones also help to maintain cellular homeostasis by triaging toxic protein aggregates, which are responsible for causing neurodegenerative diseases. Two proteins that can form these toxic aggregates are tau and α-synuclein, which form tangles in Alzheimer’s disease and Lewy bodies in Parkinson’s disease, respectively. These proteins aggregate to form small, soluble aggregates termed oligomers and long fibrils often termed amyloids, both of which are thought to be toxic. Here we show that a chaperone, cyclophilin 40 (CyP40), interacts with and dissolves tau and α-synuclein aggregates. CyP40 may accomplish this by interacting with proline residues in these proteins, which are known to play a key role in fibril stability. We show that CyP40 both lowers tau fibrils and oligomers in mice that overexpress tau protein and preserves cognition in these transgenic animals.

## Introduction

Most mammalian proteins have intrinsic sequences that promote amyloid fibril formation [1]. This is believed to be a natural process to prevent the formation of more toxic amorphous, intermediate structures [1,2]. However, in some cases such as neurodegenerative disease, aberrant amyloid formation promotes proteotoxicity [3]. Recent evidence suggests endogenous protein complexes have the ability to dissolve these potentially toxic amyloidogenic structures [4]. This work not only has shed light on novel cellular pathways of disaggregation but also suggests that these proteins could be exploited to mitigate disease pathogenesis. For example, the yeast disaggregase heat-shock protein (Hsp) 104 utilizes ATP hydrolysis to disaggregate a variety of human proteins in a yeast model [5,6]. More recently, the human Hsp70/DnaJ/Hsp110 complex was shown to facilitate the disaggregation of amyloid substrates in vitro in an ATP-dependent manner [7], but the dynamic and ubiquitous nature of aggregation and disaggregation in cells suggests that a more energy-efficient process might exist, one not dependent on ATP. However, a mammalian ATP-independent amyloid unraveling enzyme has yet to be identified.

For many proteins, amyloidogenesis is significantly affected by structurally rigid proline residues [8,9], which unlike other amino acids have a more energetically favorable *cis*-conformation [10]. This unique nature of proline residues enables major changes in protein tertiary structure through *cis-trans* isomerization [11]. In neurodegenerative disorders, such as Alzheimer’s disease (AD) and Parkinson’s disease (PD), amyloidogenic proteins (Aβ and tau in AD and α-synuclein in PD) form stacked β-sheets [10–12] containing β-turns [13]. Because proline residues, which are enriched in tau and other intrinsically disordered proteins, are frequently found in β-turns [14], we hypothesized that enzymes capable of twisting these residues could unravel amyloids.

The family of *cis*/*trans* peptidyl-prolyl isomerases (PPIases) is diverse [15] and, perhaps most importantly, does not require the use of ATP to isomerize proline residues [16]. Previous work, by our group as well as others, demonstrated that PPIases regulate tau aggregation [17–19]. Despite the fact that β-turns often contain prolines, amyloid disaggregation via PPIases has never been demonstrated. Thus, we speculated that discrete PPIases may disaggregate amyloids in a proline-dependent manner. Here, we describe the isomerase-dependent effects of human cyclophilin 40 (CyP40) in amyloid disaggregation. CyP40 is a member of the cyclophilin family, which along with FK-506 binding proteins (FKBPs) and parvulins comprise a group of proteins known as immunophilins [15]. Cyclophilins (CyPs) are defined by their ability to bind cyclosporin A (CsA). Upon binding, CyPs suppress the immune system through deactivation of calcineurin, a regulator of inflammation-inducing transcription factors [20]. CyPs also play a key role in the folding of nascent proteins by catalyzing the *cis* to *trans* conformation of proline residues, a rate-limiting step in proper folding [21,22]. Recent work has shown that the isomerase activity of CyP40 is regulated through its interaction with Hsp90 and when CyP40 is bound to Hsp90, isomerase activity is reduced. Cellular stress can release CyP40 from Hsp90, subsequently increasing CyP40 isomerase and chaperone activity [23].

In this study, we identified a novel disaggregation mechanism. For the first time, we showed that a PPIase is capable of disaggregating amyloids. We demonstrated that CyP40 exhibits disaggregation activity dependent on interactions with proline residues and independent of cofactors such as ATP. Further, 2 conformational states of CyP40, found in the Protein Data Bank, provided evidence for a potential mechanism by which amyloid binding and subsequent conformational changes in CyP40 may drive ATP-independent disaggregation activity. Transduction of CyP40 in a tauopathic brain reduced tau oligomers and tangles, yielded significant improvements in neuronal health, and preserved cognitive function. Together, these data implicate CyP40 as a potential therapeutic intervention for tauopathies and other amyloidogenic disorders.

## Results

### CyP40 disaggregates tau amyloids

**Fig 1:**
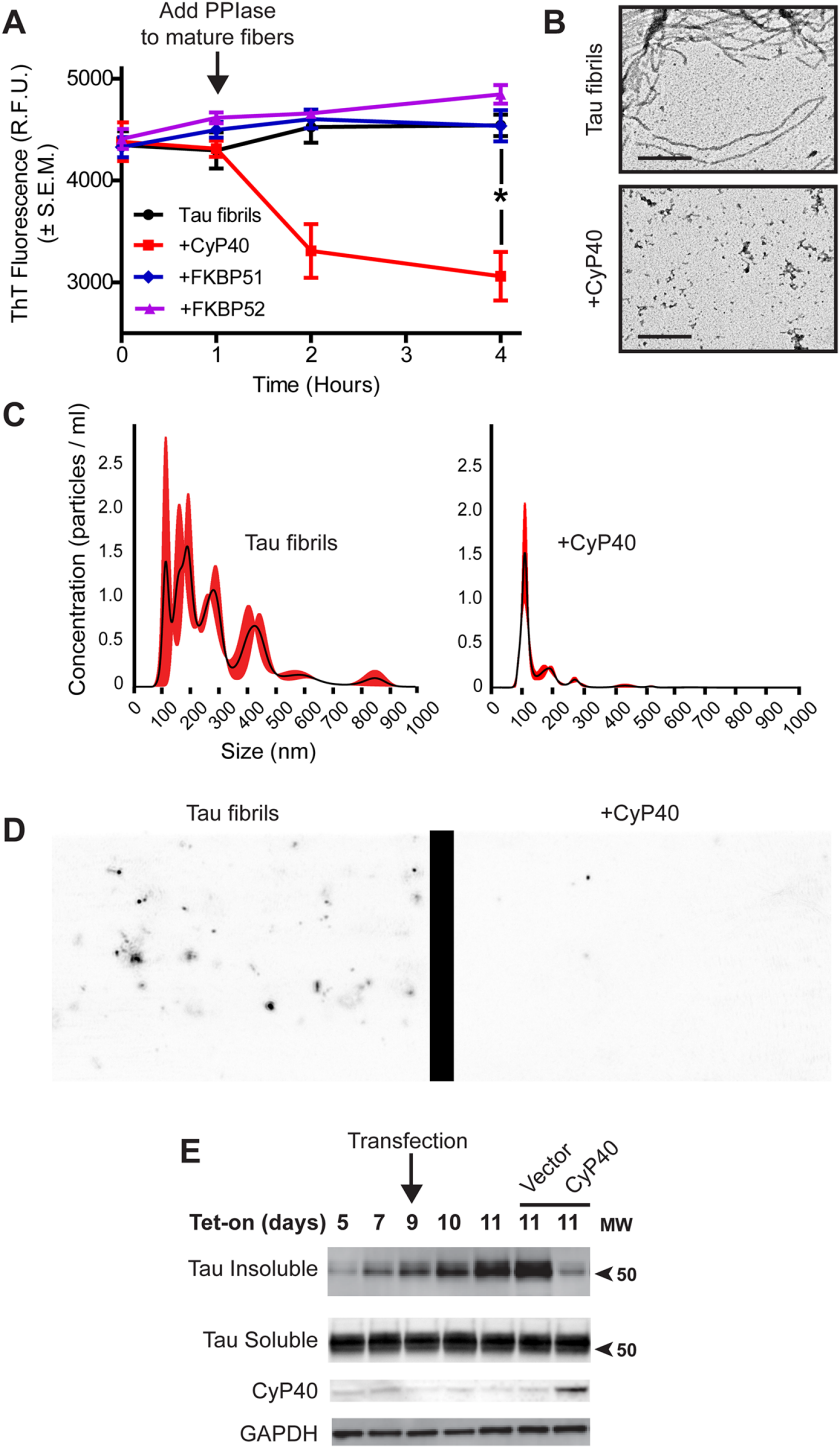
Cyclophilin 40 (CyP40) disaggregated tau fibrils. (A) Preformed tau fibrils were monitored for thioflavin T (ThT) fluorescence intensity over 4 hours. CyP40, FK-506 binding protein (FKBP) 51, and FKBP52 were added at 1 hour, and monitoring continued for 3 hours (bars represent standard error of the mean [SEM], unpaired *t* test, *p* = 0.0048, *n* = 3 independent protein preparations). (B) Representative 60,000× transmission electron microscopy (TEM) images of tau fibrils or CyP40-treated fibrils (scale bar 400 nm, *n* = 2 independent preparations of recombinant protein). (C) Nanoparticle tracking analysis (NTA) size distribution of tau fibrils and CyP40-treated tau fibrils. (D) Representative images of particles from the NTA. (E) Inducible human embryonic kidney (iHEK) cells overexpressing tau P301L (iHEK_P301L_) were induced with tetracycline on day 1 and transfected with either control vector or CyP40 on day 9. Sarkosyl-soluble and insoluble fractions were collected at day 11. Sarkosyl-insoluble fractions were probed by western blot with Tau 12 antibody. Sarkosyl-soluble fractions were probed by western blot with H-150 anti-tau, anti-CyP40, and anti–glyceraldehyde 3-phosphate dehydrogenase (GAPDH) antibodies (western blot representative of 2 biological replicates, *n* = 2). The numerical data used in panel 1A can be found in S1 Data. PPIase, peptidyl-prolyl isomerase; RFU, relative fluorescence unit.

To investigate the activity of PPIases on tau fibrils, we purified 3 Hsp90 cochaperones that possess PPIase activity: CyP40, FKBP51, and FKBP52. Individual PPIases were incubated with fibrillized recombinant P301L tau for 3 hours, and thioflavin T (ThT) fluorescence was measured periodically. CyP40 decreased ThT fluorescence, indicating a reduction in β-sheet secondary structure (Fig 1A). It should be noted that the ThT signal was not entirely ablated, indicating that the tau species formed still maintain beta-sheet content. An additional ThT assay showed that this effect was concentration dependent, since increasing the molar ratio of CyP40 to tau fibrils further decreased ThT fluorescence (S1 Fig). Moreover, we determined that tau did not reaggregate after CyP40 was inactivated by CsA (S2 Fig). Furthermore, FKBP51 and FKBP52, despite having a more robust PPIase activity relative to CyP40 (S3 Fig), did not lower amyloid content. This is possibly due to differential substrate binding between tau and PPIases, as will be discussed later.

We then further characterized the tau species formed following incubation with CyP40. CyP40 dramatically altered the morphology and reduced the size of tau fibrils as observed by transmission electron microscopy (TEM) (Fig 1B). We then utilized nanoparticle tracking analysis (NTA) to analyze the particle size profile of recombinant tau fibrils in the presence of CyP40. While mature tau P301L fibrils exist as an array of particles up to 850 nm in size, CyP40-coincubated samples lacked particles greater than 150 nm in length (Fig 1C and 1D), corroborating the results of the ThT and TEM assays. CyP40 alone showed a minimal number of particles (S4 Fig). To further confirm CyP40 disaggregase activity, we utilized an inducible tau cell model [24] capable of forming insoluble tau. Sarkosyl-insoluble tau was reduced by the overexpression of CyP40 (Fig 1E). An AlamarBlue assay showed that CyP40 was not toxic in the inducible human embryonic kidney (iHEK_P301L_) model (S5 Fig), confirming insoluble tau reduction was not caused by CyP40-associated toxicity. Taken together, these data indicate that CyP40 is capable of disaggregating amyloid fibrils in vitro.

### CyP40 converted tau from sarkosyl-insoluble to sarkosyl-soluble in vivo

**Fig 2:**
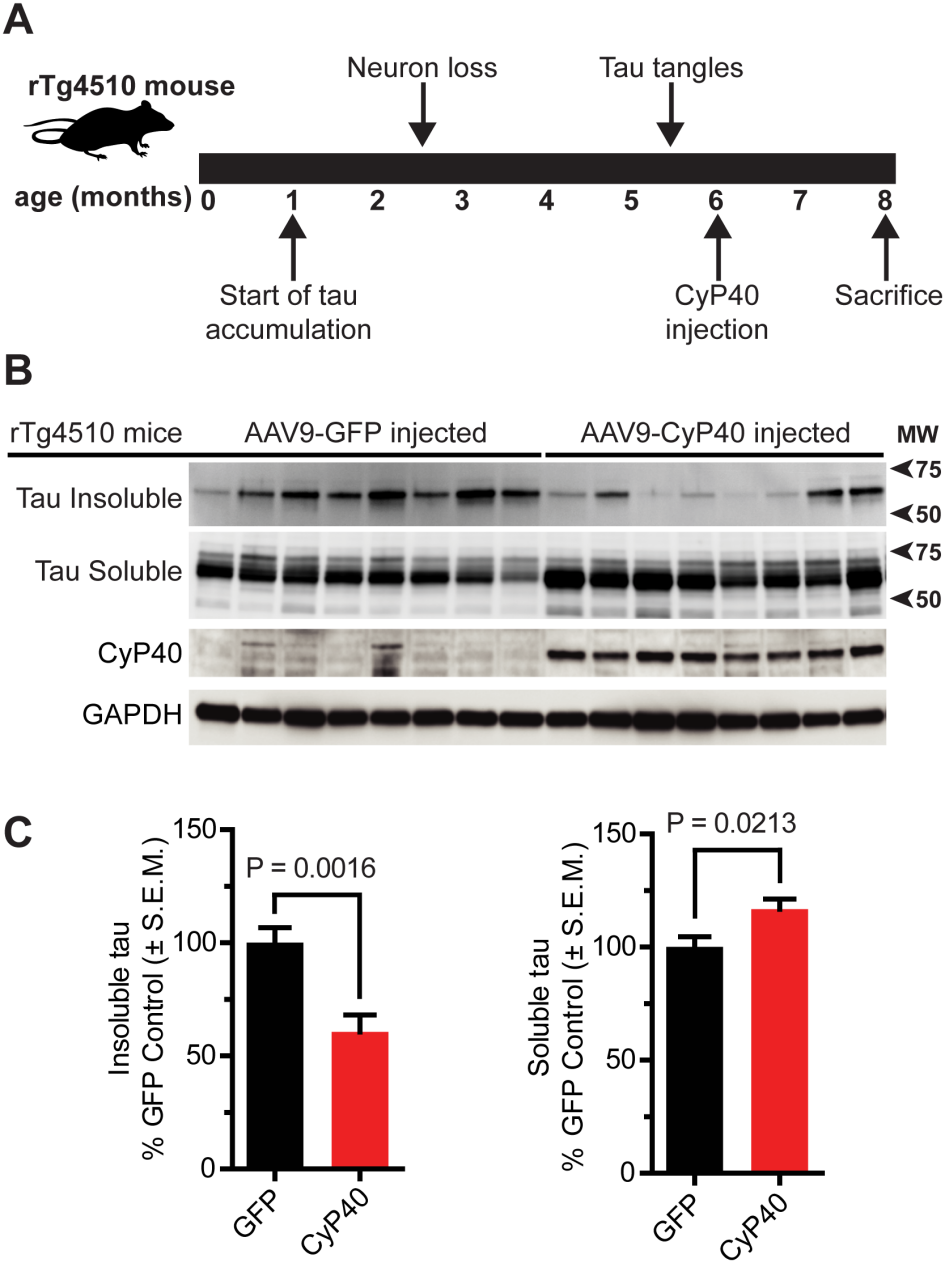
Cyclophilin 40 (CyP40) overexpression decreased sarkosyl-insoluble tau. (A) Schematic depicting the timeline of rTg4510 tau pathology and experimental design. (B) Western blot comparing sarkosyl-insoluble and sarkosyl-soluble fractions from the hippocampi of adeno-associated virus serotype 9–green fluorescent protein (AAV9-GFP) (*n* = 8) and AAV9-CyP40 (*n* = 8) injected rTg4510 mice. Each lane indicates an individual transgenic mouse. (C) Quantification of the relative insoluble and soluble tau for AAV9-GFP and AAV9-CyP40 injected mice (unpaired *t* test, *n* = 8 for each group). The numerical data used in panel 2C can be found in S1 Data. GAPDH, glyceraldehyde 3-phosphate dehydrogenase; SEM, standard error of the mean.

The capacity of CyP40 to lower insoluble tau levels was then evaluated in a mouse model of tauopathy. Hippocampi of rTg4510 mice were injected with adeno-associated virus serotype 9–green fluorescent protein (AAV9-GFP) or AAV9-CyP40 at 6 months of age and were harvested at 8 months for biochemical and histological analyses (Fig 2A, S6A Fig). These ages were chosen so that the disaggregation activity of CyP40 on insoluble tau fibrils could be investigated. CyP40 and GFP protein expression was achieved throughout the hippocampus (S6B Fig). CyP40 was expressed 9.2× higher in mice transduced with AAV9-CyP40 compared to GFP-injected control mice (S7 Fig). CyP40 overexpression caused a significant decrease in the amount of sarkosyl-insoluble tau while significantly increasing soluble tau levels (Fig 2B and 2C), suggesting CyP40 disaggregated insoluble tau in vivo.

### CyP40 reduced pathologically relevant tau and preserved neuron viability

**Fig 3:**
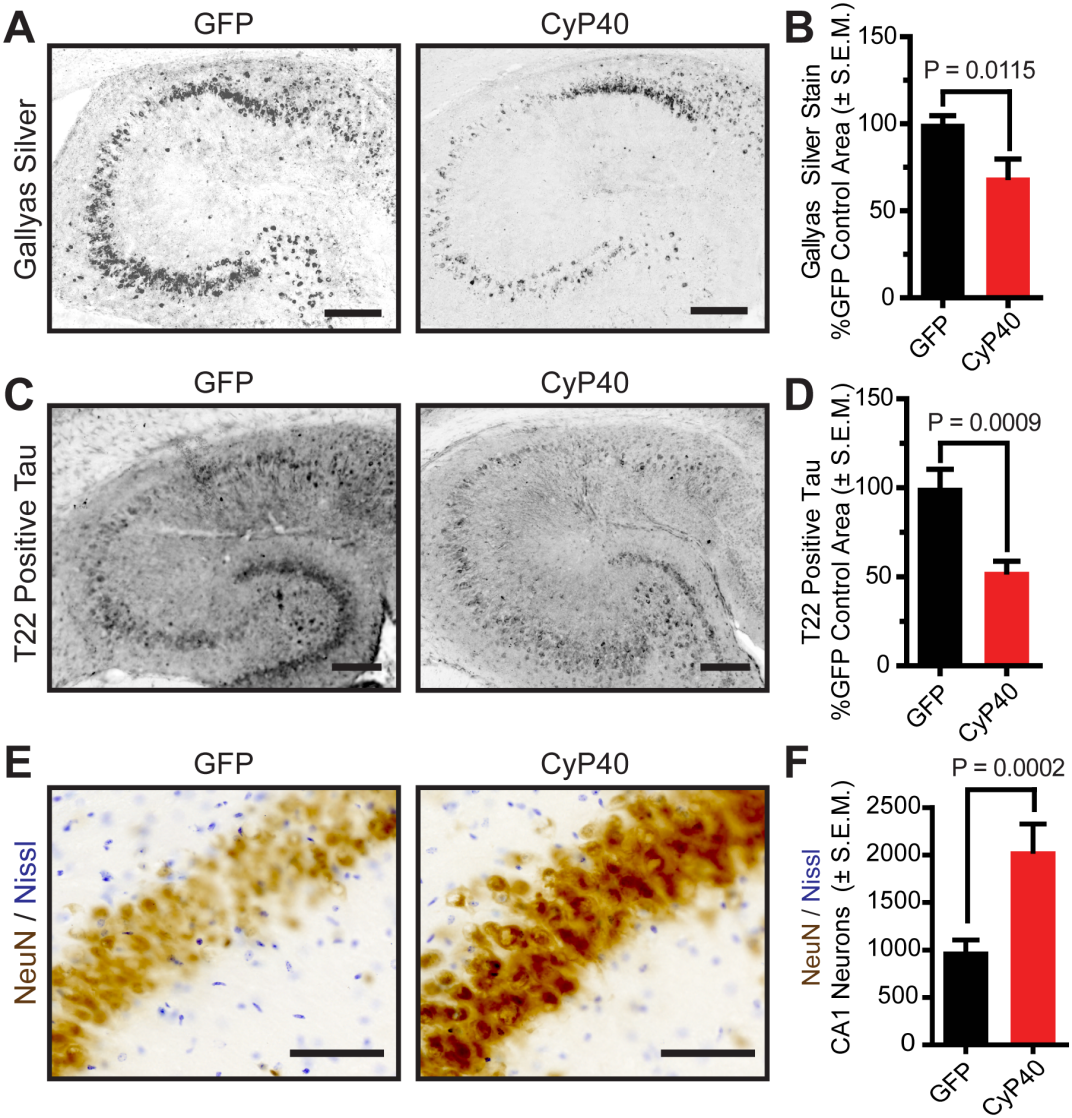
Cyclophilin 40 (CyP40) reduced tau deposits and preserved hippocampal CA1 neurons in vivo. (A) Representative images of Gallyas silver-stained hippocampi from adeno-associated virus serotype 9–green fluorescent protein (AAV9-GFP) and AAV9-CyP40 injected mice (scale bar 200 μm). (B) Quantification of Gallyas silver-staining (unpaired *t* test, *n* = 8 for each group) (C) Representative images of T22-oligomeric tau stained hippocampi from AAV9-GFP and AAV9-CyP40 injected mice (scale bar 200 μm). (D) Quantification of T22-oligomeric tau staining (unpaired *t* test, *n* = 8 for each group) (E) Representative images of CA1 neurons stained with NeuN (brown) and Nissl (violet) are shown (scale bar 125 μm). (F) Neuron counts from unbiased stereology of CA1 neurons in hippocampal sections from AAV9-GFP and AAV9-CyP40 injected mice (unpaired *t* test, *n* = 8 for each group). The numerical data used in panels 3B, 3D, and 3F can be found in S1 Data. SEM, standard error of the mean.

To further characterize the state of tau following CyP40 overexpression, immunohistochemical analyses were conducted. Transduction of CyP40 significantly reduced the levels of Gallyas silver-positive tau tangles (Fig 3A and 3B), a hallmark of tauopathies [25,26]. However, it has been suggested that soluble tau oligomers, like those recognized by T22 tau antibody [27], are more closely associated with neurodegeneration than tau tangles [28–31]. To determine if the transition of tau from insoluble to soluble was producing toxic oligomers, we quantified the levels of T22-positive tau. CyP40 decreased T22-positive tau levels (Fig 3C and 3D), demonstrating that CyP40 can affect numerous multimeric tau species regardless of solubility. To determine if Cyp40 overexpression was neuroprotective, neuron counts were performed using unbiased stereology. CyP40 overexpression significantly preserved CA1 neurons (Fig 3E and 3F), suggesting that CyP40 may disaggregate tau into nontoxic species.

### CyP40 overexpression rescued tau-induced cognitive deficits

**Fig 4:**
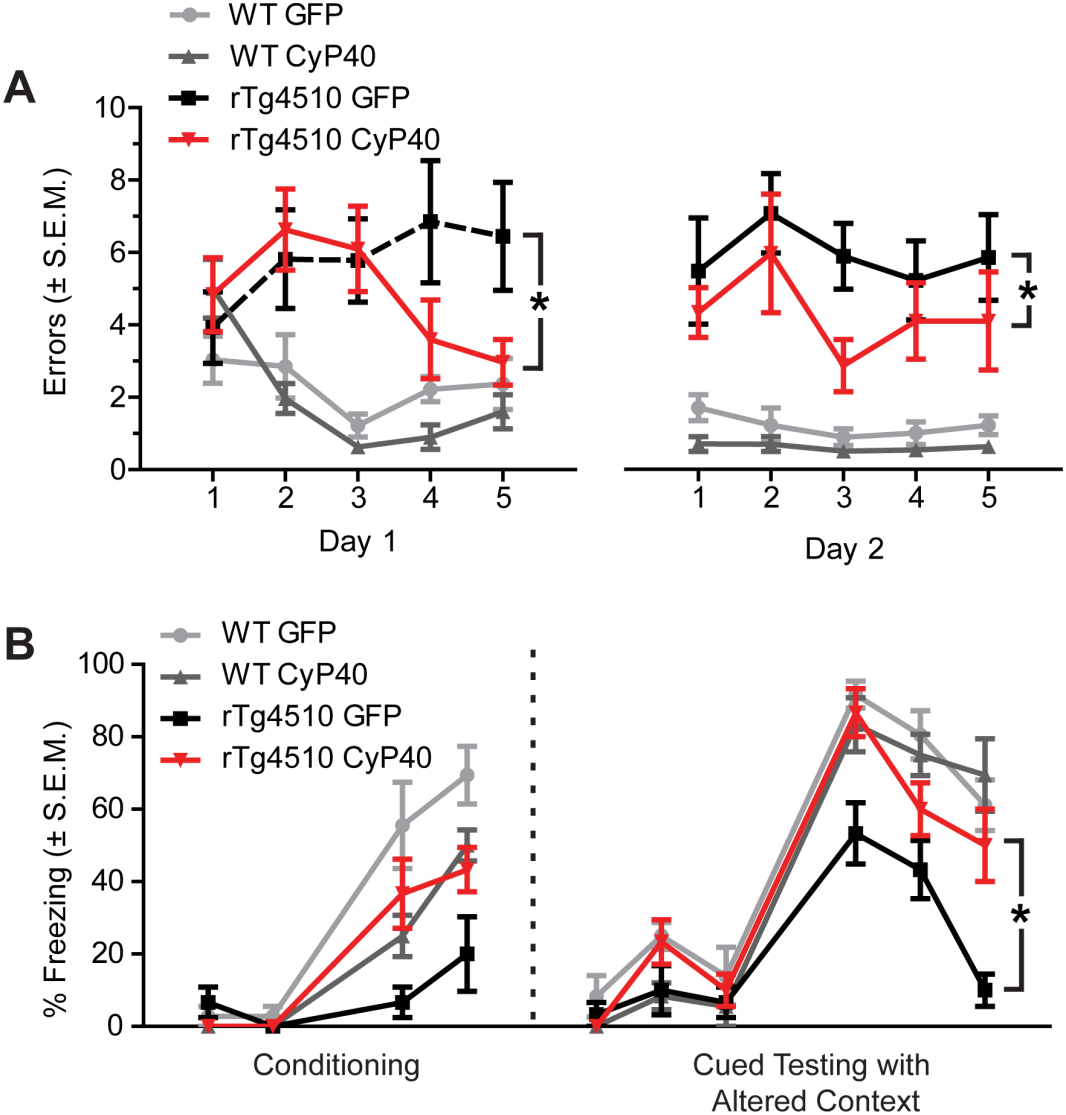
Cyclophilin 40 (CyP40) rescued tau-induced cognitive deficits. (A) Radial-arm water maze spatial memory analysis of adeno-associated virus serotype 9–green fluorescent protein (AAV9-GFP) injected wild-type (wt) (*n* = 9), AAV9-CyP40 injected wt (*n* = 10), AAV9-GFP injected rTg4510 mice (*n* = 9), and AAV9-CyP40 injected rTg4510 mice (*n* = 10). Data were analyzed by two-way ANOVA, and the significant results were followed by Tukey post hoc tests (*p < 0.05). (B) Percent freezing in the cued fear conditioning task (*n* = 6 for each wt group and *n* = 5 for each transgenic group). Data were analyzed by two-way ANOVA, and the significant results were followed by Tukey post hoc tests (*p < 0.05). The numerical data used in figure can be found in S1 Data. SEM; standard error of the mean.

To evaluate the effects of CyP40 overexpression on learning and memory, rTg4510 and wild-type (wt) mice were injected with AAV9-GFP or AAV9-CyP40 at 3 months of age. CyP40-overexpressing rTg4510 mice showed a significant reduction in errors compared to GFP-overexpressing rTg4510 mice in the 2-day radial-arm water maze paradigm (Fig 4A). This reduction in errors suggests that spatial learning and memory is more intact in rTg4510 mice overexpressing CyP40 [32]. Significant differences were not found in CyP40- or GFP-overexpressing WT mice. Additionally, CyP40 overexpression in rTg4510 mice rescued tau-induced freezing deficits in the cued fear conditioning task (Fig 4B). This increase in freezing is indicative of a preservation of associative learning and memory [33], suggesting CyP40 disaggregated existing tau fibrils or prevented the accumulation of aggregating tau prophylactically. Collectively, these data suggest that CyP40 decreased tau aggregates, reduced toxic tau oligomers, increased neuronal survival, and improved cognitive function in vivo.

### CyP40 disaggregates α-synuclein, but not β-amyloid

**Fig 5:**
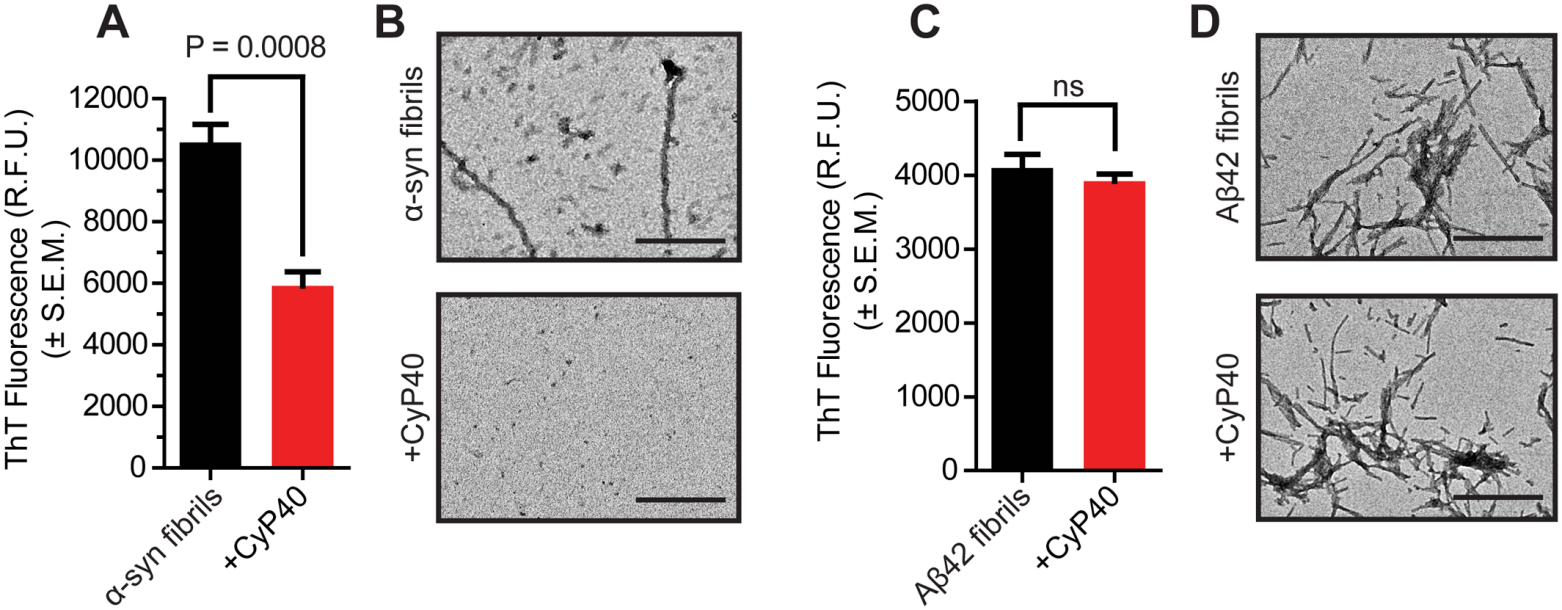
Cyclophilin 40 (CyP40) disaggregated α-synuclein, but not amyloid_1-42_ peptide (Aβ42) fibrils. (A) Thioflavin T (ThT) fluorescence of A53T α-synuclein (α-syn) fibrils ± CyP40 protein (unpaired *t* test, *n* = 4). (B) Representative transmission electron microscopy (TEM) images of α-syn A53T fibrils ± CyP40 (60,000× magnification; scale bar 400 nm) (C) ThT fluorescence of Aβ42 fibrils ± CyP40 protein (unpaired *t* test, *n* = 4). (D) Representative TEM images of Aβ42 fibrils ± CyP40 (60,000× magnification; scale bar 400 nm). The numerical data used in panels 5A and 5C can be found in S1 Data. RFU, relative fluorescence unit; SEM, standard error of the mean.

To investigate the potential for CyP40 to disaggregate amyloids other than tau, we generated recombinant fibrils of A53T α-synuclein (α-syn) and β-amyloid_1-42_ (Aβ42). Similar to tau, CyP40 decreased ThT fluorescence when incubated with α-syn fibrils (Fig 5A); TEM and NTA corroborated these results (Fig 5B, S8A and S8B Fig). Conversely, CyP40 was unable to disaggregate Aβ42 fibrils (Fig 5C and 5D, S8C and S8D Fig). Both tau and α-syn contain proline residues; Aβ42 does not. This suggests CyP40 disaggregation activity is proline dependent, thus supporting a PPIase-dependent mechanism.

### CyP40 selectively binds proline-containing regions of tau and α-syn

**Fig 6:**
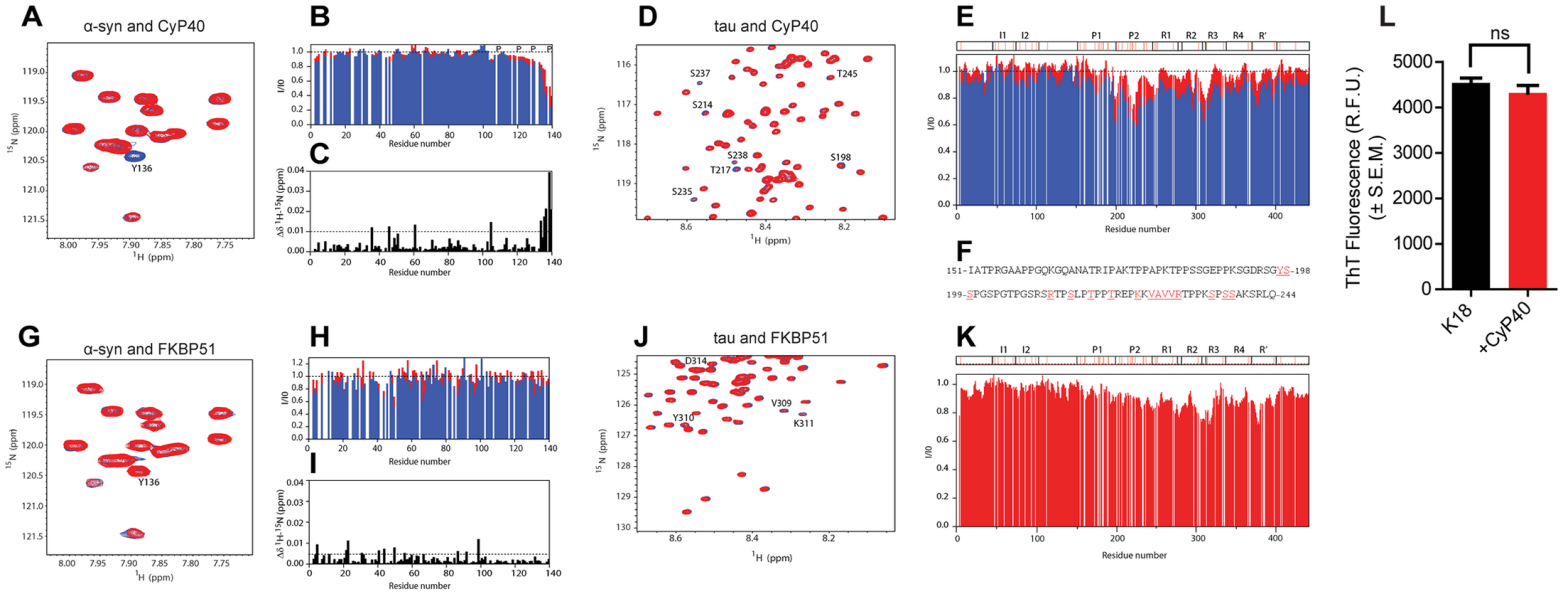
Cyclophilin 40 (CyP40) binds proline-containing regions of tau and α-synuclein (α-syn). (A) A selected region of the 2D [^1^H-^15^N]–heteronuclear single quantum coherence (HSQC) experiment of ^15^N-labeled α-syn before (blue) and after (red) addition of CyP40 (molar ratio 1:15). Y136 is marked. (B) Normalized residue-specific nuclear magnetic resonance (NMR) intensities in the presence of a 5-fold (red) and 15-fold (blue) excess of Cyp40; the location of proline residues is indicated. (C) Combined [^1^H-^15^N] chemical shift perturbation analysis upon addition of CyP40 to α-syn. (D) A selected region of 2D [^1^H-^15^N]-HSQC experiment of tau before (blue) and after (red) addition of CyP40 (molar ratio 1:10) is shown. Residues displaying significant intensity loss are labeled. (E) Normalized residue-specific NMR intensities of tau in the presence of a 5-fold (red) and 10-fold (blue) excess of CyP40; tau’s domain organization is shown, and proline residues are marked with red. (F) Amino acid sequence of the proline-rich regions P1 (top) and P2 (bottom); residues significantly broadened upon addition of CyP40 are colored red. (G) A selected region of the 2D [^1^H-^15^N]-HSQC experiment of ^15^N-labeled α-syn before (blue) and after (red) addition of FK-506 binding protein (FKBP) 51 (molar ratio 1:10) is displayed. (H) Normalized residue-specific NMR intensities in the presence of a 5-fold (red) and 10-fold (blue) excess of FKBP51. (I) Combined [^1^H-^15^N] chemical shift perturbation analysis upon addition of FKBP51 to α-syn. (J) Selected region of a 2D [^1^H-^15^N]-HSQC experiment of tau before (blue) and after (red) addition of FKBP51 (molar ratio 1:10). Final concentrations of CyP40 and FKBP51 for NMR experiments are 200 μM. (K) Tau residue-specific NMR intensities in presence of a 10-fold excess of FKBP51. (L) Thioflavin T fluorescence of K18 tau aggregates in the presence or absence of CyP40 (unpaired *t* test, *p* > 0.05, *n* = 3). The numerical data used in panel 6L can be found in S1 Data.

Next, we acquired 2D heteronuclear single quantum coherence (HSQC) spectra of ^15^N-labeled α-syn and tau in the presence and absence of unlabeled CyP40. This provides a spectral signature encompassing all amide bonds within a protein, allowing us to map interaction sites by recording variations in position and/or intensity of individual peaks. Upon Cyp40 addition to α-syn, the residues present at the C-terminus of the protein displayed a significant intensity loss together with changes in NMR signal position (Fig 6A–6C). In the case of tau, a subset of signals mostly contained in the proline-rich region were attenuated with increasing CyP40 concentration (Fig 6D–6F), indicating that the binding between the 2 proteins is specific to this region. The reduction in signal intensity in protein NMR is typically caused by the combined effects of an increase in molecular weight due to complex formation, which accelerates transverse relaxation and chemical exchange at the contact surface [34–36]. The reduction in intensity seen here is consistent with an intermediate exchange time scale of CyP40-tau binding. Based on these findings, CyP40 selectively recognized proline-rich regions in both proteins. Interestingly, proline-containing β-turn structures are thought to be exposed in mature fibrils [37], providing a possible mechanism for how CyP40 accomplishes its disaggregation function. Conversely, HSQC spectra of both proteins in the presence of FKBP51 indicate that FKBP51 does not interact with monomeric α-syn (Fig 6G–6I) and suggest that FKBP51 binds to the microtubule binding region of tau instead of proline-rich domains (Fig 6J and 6K). This offers a possible explanation for the lack of FKBP51 disaggregation activity relative to CyP40. To further support the need for CyP40 binding to the proline-rich region of tau in order to disaggregate amyloid fibrils, CyP40 was incubated with fibrillized K18 fragment of tau consisting of only the microtubule-binding domain repeats. CyP40 was unable to disaggregate this substrate (Fig 6L).

### CyP40-mediated disaggregation is diminished in a PPIase-null CyP40 mutant

**Fig 7:**
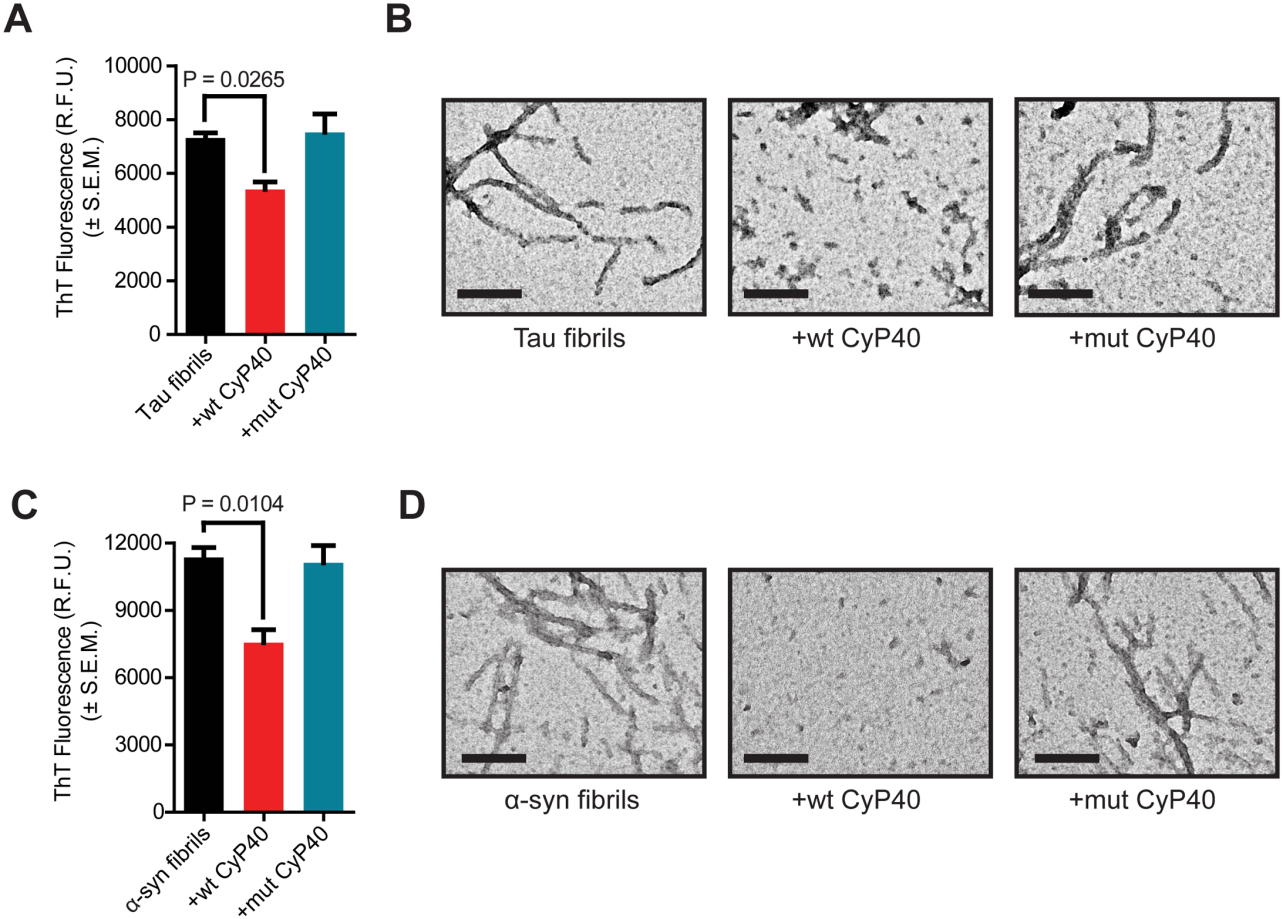
Cyclophilin 40 (CyP40)-mediated disaggregation assay of wild-type (wt) and peptidyl-prolyl isomerase (PPIase) null mutant (mut) CyP40. (A) Thioflavin T (ThT) fluorescence of tau ± wt CyP40 or mut CyP40 (unpaired *t* test, *n* = 3 for each condition). (B) Representative transmission electron microscopy (TEM) images of (A) (scale bar 200 nm). (C) ThT fluorescence of A53T α-synuclein (α-syn) ± wt CyP40 or mut CyP40 (unpaired *t* test, *n* = 3 for each condition). (D) Representative TEM images of (C) (scale bar 200 nm). The numerical data used in panels 7A and 7C can be found in S1 Data. RFU, relative fluorescence unit; SEM, standard error of the mean.

To further explore the mechanism of CyP40-mediated disaggregation, a mutant of CyP40 lacking PPIase activity was constructed. Mutation of histidine 141 to glutamate (H141E) ablated PPIase activity as shown by a chymotrypsin-coupled assay (S9A Fig). Importantly, the mutation did not significantly alter the secondary structure of the protein as evidenced by circular dichroism (S9B Fig). Wild-type (wt) and mutant (mut) CyP40 were incubated with tau P301L fibrils, and ThT fluorescence was recorded. Cyp40 lacking PPIase activity was unable to disaggregate tau fibrils (Fig 7A). Corresponding TEM showed fibril morphology was maintained following incubation with mut CyP40, while, as expected, wt CyP40 decreased fibrils (Fig 7B). Similarly, CyP40 PPIase activity was essential for A53T α-syn fibril disaggregation (Fig 7C and 7D). Collectively, these data indicate that the PPIase activity of CyP40 is necessary for disaggregation activity in vitro. These findings also rule out the possibility that the disaggregation activity of CyP40 was the result of any technical artifacts caused by production or purification of recombinant proteins [38].

**Fig 8:**
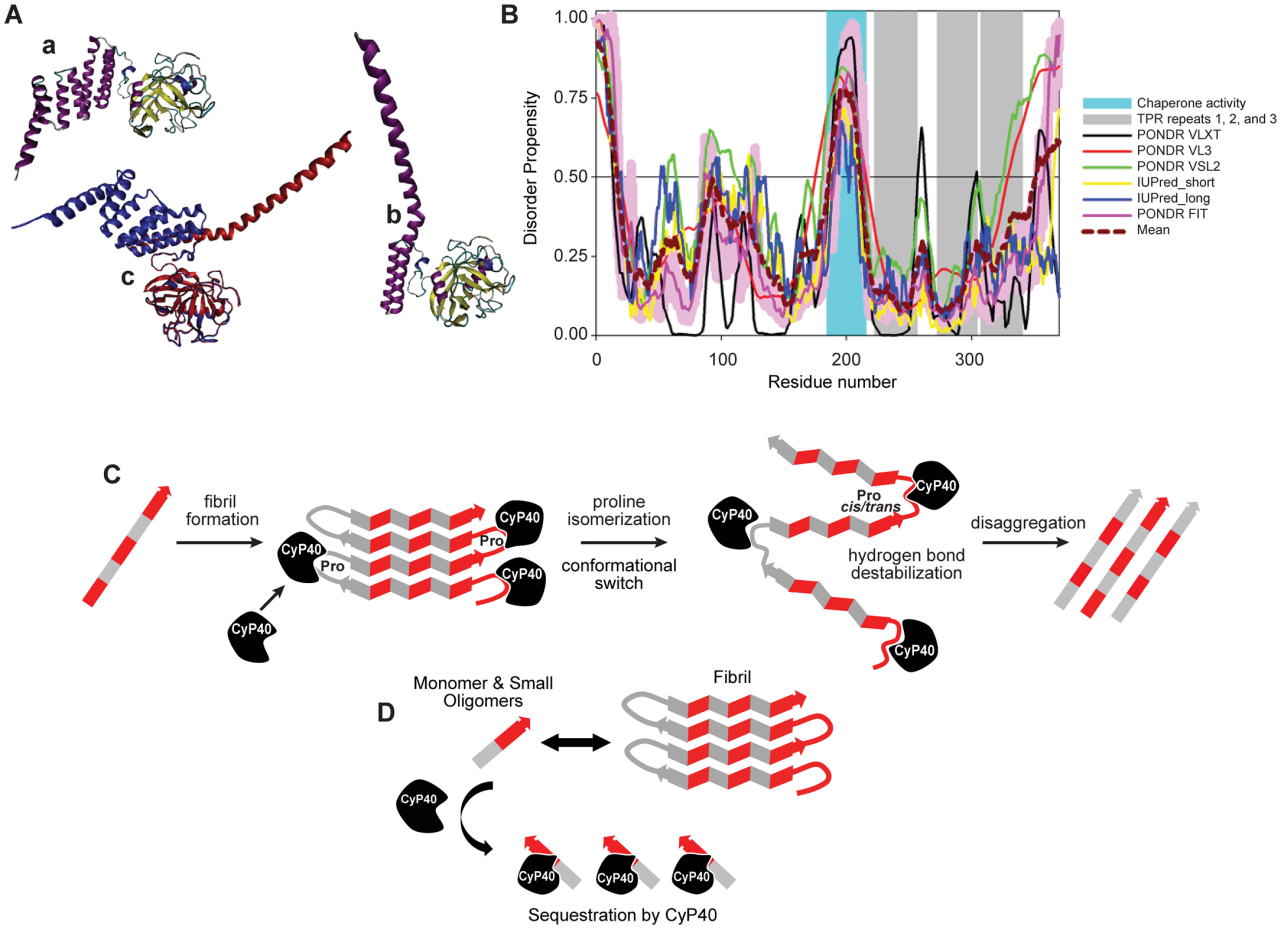
Cyclophilin 40 (CyP40) conformation, disorder prediction, and putative disaggregase mechanisms. (A) Structural characterization of bovine CyP40: a, crystal structure of the monoclinic form of CyP40 (Protein Data Bank [PDB] ID: 1ihg); b, crystal structure of the tetragonal form of CyP40 (PDB ID: 1iip). Structures in plots a and b are colored according to the secondary structure content; c, multiple structural alignment of the monoclinic (blue) and tetragonal (red) forms of CyP40 conducted by MultiProt platform. (B) Evaluating intrinsic disorder propensity of human CyP40 (UniProt ID: Q08752) by a series of per-residue disorder predictors. Disorder profiles generated by Predictor of Naturally Disordered Regions (PONDR) VLXT, PONDR VL3, PONDR VSL2, IUPred_short, IUPred_long, and PONDR FIT are shown by black, red, green, yellow, blue, and pink lines, respectively. The light pink shadow around the PONDR FIT shows error distribution. The cyan-shaded area shows the position of the region with chaperone activity. The positions of 3 tetracopeptide repeat (TPR) motifs are shown as gray-shaded areas. In these disorder analyses, the predicted intrinsic disorder scores above 0.5 are considered to correspond to the disordered residues/regions. (C) Schematic representation of hypothesized enzymatic activity-based CyP40 disaggregation. (D) Schematic representation of hypothesized interaction-based CyP40 disaggregation.

We further explored the putative molecular mechanism of CyP40-mediated disaggregation through the analysis of its intrinsic disorder propensity and through available structural information. Structural data in the Protein Data Bank (PDB) are available only for bovine CyP40, which shares 93.78% sequence identity along with similar disorder plot profiles to human CyP40 (S10 Fig). Structural analysis of bovine CyP40 revealed that this protein can be crystallized in the monoclinic and tetragonal forms. These forms differ dramatically in the spatial organization of the C-terminal domain containing 3 tetratricopeptide repeat (TPR) motifs (residues 223–256, 273–306, and 307–340) [39]. Fig 8Aa shows that although the monoclinic form is characterized by the presence of 7 α-helices of variable length (residues 216–235, 239–259, 269–285, 289–300, 307–319, 323–336, and 341–362) packed in a relatively compact domain (PDB ID: 1ihg), the structure of the tetragonal form (PDB ID: 1iip) is remarkably different (Fig 8Ab). Fig 8Ac shows that the structures of PPIase cyclophilin-type domain and TPR-1 motif remain mostly unperturbed. In fact, in multiple structural alignment, the root-mean-square deviation (RMSD) for this region spanning residues 1–259 is remarkably low: 0.53 Å. On the other hand, 2 α-helices of TPR-2 have straightened out and, together with the second helix of the TPR-1, form 1 extended α-helix (residues 239–297) that protrudes out from the protein. Furthermore, this structure does not contain coordinates for the C-terminal region of this protein (residues 298–369), which includes the TPR-3 and the C-tail containing a putative calmodulin-binding site [39]. Since the C-terminal half of CyP40 (residues 185–370) is responsible for the chaperone activity as well as the interaction with Hsp90 and several other binding partners, it was suggested that a partially folded form of the TPR-containing domain may be related to the functionality of this protein [39]. The ability of CyP40 to undergo dramatic conformational changes from a more closed, monoclinic-like structure to a widely open, tetragonal-like form (see Fig 8A) potentially resides in the specifics of the intrinsic disorder propensity distribution within its sequence. In agreement with this hypothesis, Fig 8B shows that regions linking the PPIase and TPR-containing domains and linkers between the individual TPR motifs are predicted to be either high disordered (have a disorder score above 0.5) or at least flexible (have disorder scores between 0.25 and 0.5), thereby providing means for high conformational flexibility. Though the exact mechanism by which CyP40 disaggregates fibrils remains unclear, this is the first demonstration of a single human protein to display disaggregase activity both in vitro and in vivo.

## Discussion

In this study, we describe the disaggregation activity of the human cyclophilin, CyP40. This is the first description of a PPIase disaggregating neurodegenerative amyloids, such as tau and α-syn. This disaggregation is dependent on the PPIase activity of CyP40 and is ATP independent. The distinct structural properties of CyP40 may enable this ATP-independent process to occur and thus may be unique to CyP40 among other PPIases. Disaggregation of tau fibrils by CyP40 reduced pathogenic tau, concomitant with decreased neuronal toxicity and increased cognitive performance.

While being the first human PPIase to display disaggregation activity, CyP40 is not the first disaggregase to be identified. Certain chaperone complexes have been shown to facilitate the disaggregation of oligomers and fibrils [4,38,40]. For example, the Hsc70/DNAJB1/Apg2 containing complex was shown to reverse the formation of α-syn fibrils in vitro. Identification of this complex was notable because, while previous attempts at chaperone-mediated amyloid disassembly required weeks of incubation in vitro [13], this complex solubilized α-syn fibrils in under an hour [4]. Disaggregation by the Hsc chaperone complex required ATP; however, our study demonstrated that fibrils could be disaggregated with similar kinetics by CyP40 in the absence of ATP. Regardless of energy dependence, the existence of amyloid disaggregases presents a new avenue for therapeutic strategies. The procognitive effects of CyP40 overexpression in the tauopathic brain suggest that strategies to either induce or deliver disaggregases to the central nervous system could halt or even rescue cognitive deficits associated with neurotoxic amyloids.

We hypothesize 2 possible mechanisms for amyloid disaggregation by CyP40. Based on our structural data (Fig 8A), it is possible that the PPIase domain interacts with specific proline residues in β-turns and twists these residues. Given that CyP40-mediated disaggregation occurs without the input of external energy, it is possible that the disaggregation reaction energy comes from the PPIase domain of CyP40 binding to key residues within the amyloid substrate followed by a conformational switch within the TPR domain (Fig 8C). There is precedent for disaggregation independent of external energy as described for the plant chaperone, cpSRP43 [41]. Because of the equimolar amount of substrate and CyP40 used in the ThT-monitored disaggregation experiments, an alternative mechanism could be proposed. In this hypothesis, CyP40 may interact with and sequester small oligomers and monomers away from the aggregation equilibrium (see Fig 8D). However, this hypothesis does not explain the lack of disaggregation activity in the PPIase-null CyP40. Therefore, further experiments are required to determine the precise mechanism by which CyP40 disaggregation occurs. It should be noted that the 2 proposed models become the same if CyP40 remains bound to the substrate.

Though CyP40 can directly interact with Hsp90 through a TPR domain, the effects described here do not appear to be Hsp90 dependent. In fact, the PPIase activity of CyP40 is reduced upon interaction with Hsp90 [23]. This may explain why tau fibrils were only disaggregated upon the overexpression of CyP40. Further studies are required to determine if there are endogenous mechanisms to increase CyP40 activity either through the up-regulation of CyP40 expression, by increasing CyP40 stability, or by increasing the enzymatic activity of CyP40. Recent work suggests that upon cellular stress Hsp90 may dissociate from CyP40, leading to an increased pool of more catalytically active CyP40 [23]. This may hint at a possible mechanistic pathway by which CyP40 may be “turned on” in response to stress, including toxic amyloid build-up.

In addition to CyP40, there are currently 41 known human PPIases within the cyclophilin, FKBP, and parvulin families. Therefore, future screening may reveal additional PPIases with activities similar to CyP40, including ATP-independent disaggregation. Additionally, CyP40 and other PPIases should be further characterized for disaggregation activity against proline-containing amyloids, especially those associated with disease.

## Materials and methods

### Ethics statement

All experimental animal procedures were performed according to our use protocol, R3103, which was approved and issued by the University of South Florida’s Institutional Animal Care and Use Committee (IACUC). The university has a PHS Animal Welfare Assurance #A-4100-01 on file with the Office of Laboratory Animal Welfare, Public Health Service, and maintains research facility registration #58-R-0015 with the United States Department of Agriculture, Animal and Plant Health Inspection Service.

### Protein purification

Recombinant human tau P301L, CyP40, CyP40 H141E, FKBP51, and FKBP52 were cloned into bacterial expression vectors, and protein was expressed in *E*. *coli* BL21 cells, purified, and dialyzed. Proteins were estimated to be >95% pure by Coomassie staining. The details are given in S1 Text. Amyloid beta protein fragment 42 was purchased from Sigma Aldrich (107761-42-2), and mature α-syn fibrils were generated according to the established protocol by shaking the α-syn solution, at a concentration of 0.3 mg/ml (20.8 μM), in 10 mM HEPES buffer (pH 7.5) and 100 mM NaCl.

### Fibril formation

Fifty μM recombinant human tau P301L was incubated in the presence of 12.5 μM low-molecular-weight heparin at 37°C in 100 mM Sodium Acetate pH 7.0 buffer without agitation for 7 days. Aβ42 fibrils were formed by suspending the lyophilized protein in minimal 60 mM NaOH and then diluting to 50 μM in PBS buffer. The solution was then shaken at 700 rpm at 37°C for 5 days. ThT fluorescence was recorded throughout fibrillation in order to confirm a plateau of fluorescence.

### ThT fluorescence assay

Pre-fibrillized tau (7.5 μM) was incubated with 7.5 μM CyP40 (wt or mut where indicated) protein (in 100 μM Sodium Acetate pH 7.0 buffer for tau or PBS for α-syn and Aβ42) in 100 μL volumes in a 96-well, black, clear-bottom plate (Fisher #07-200-525). At set time points (0, 1, and 3 hours), 7.5 μM ThT (final concentration) was added and fluorescence read at 440 nm excitation and 482 nm emission in a BioTek Synergy H1 plate reader. Experiments were performed in at least duplicate.

### TEM

Ten μL of protein samples was adsorbed onto square mesh copper grids (EMS300-Cu) for 30 seconds. Grids were washed twice with 10 μL of deionized water, and the excess water was removed. Samples were negatively stained with 4% uranyl acetate for 30 seconds and dried overnight. Grids were viewed using a JEOL 1400 Digital Transmission Electron Microscope, and images were captured with a Gatan Orius wide-field camera. The fields shown are representative.

### NTA

Samples were diluted 10,000-fold into 1 mL of 0.02 μm filtered deionized water. Approximately 300 μL of sample was loaded onto the Malvern Nanosight LM10 equipped with a 633 nm red laser. Protein particle data were captured with a Marlin CCD camera in duplicate. Graphs were generated by Nanosight software.

### Circular dichroism (CD) spectroscopy

Far-UV CD measurements were taken using a JASCO J-815 spectropolarimeter. wt CyP40 and mut CyP40 were dialyzed into 10 mM sodium phosphate buffer (pH 7.5), and readings were taken at 25°C. Each spectrum is an average of 3 scans from 190–260 nm at 50 nm/min. Buffer spectral curves were subtracted for each protein.

### NMR spectroscopy

^15^N-labeled human tau and α-syn proteins were expressed and purified as described previously [42,43]. NMR samples contained 15 μM ^15^N single-labeled α-syn protein in 50 mM HEPES buffer (pH 6.8), 100 mM NaCl, 0.02% NaN_3_, and 10% (v/v) D_2_O or 20 μM ^15^N single-labeled tau protein in 50 mM phosphate buffer (pH 6.8), 0.02% NaN_3_, and 10% (v/v) D_2_O. Unlabeled CyP40 and FKBP51 samples were dialyzed in the same buffers. NMR experiments of α-syn and tau were recorded at 15°C and 5°C, respectively, on 600, 700, 800, and 900 MHz spectrometers (Bruker) equipped with cryogenic or room-temperature probes. Final concentrations of CyP40 and FKBP51 were 200 μM, while tau was 20 μM. Two-dimensional ^1^H–^15^N HSQC spectra were acquired in the case of α-syn using 256 complex points in the indirect dimension and 64 increments with spectral widths of 8,417.5 and 1,776.1 Hz in the ^1^H and ^15^N dimensions or with 512 complex points in the indirect dimension and 40 scans with spectral widths of 8,417.5 and 1,705.1 Hz in the case of tau. Spectra were processed with NMRPipe [44] and analyzed using Sparky (T. D. Goddard and D. G. Kneller, SPARKY 3, University of California, San Francisco). NMR intensity ratio plots were reported with a 3-residues averaging window.

### Chymotrypsin-coupled assay

PPIase activity was measured by a chymotrypsin-coupled assay using a synthetic peptide succinyl-Ala-Ala-Pro-Phe-p-nitroanilide (Sigma) as previously described [51] with some modifications. Briefly, the reactions were set up in precooled 1 ml reaction buffer (50 mM Tris-HCl, pH 7.5, 150 mM NaCl, 10 mM CaCl_2_) by adding 5 μl 1 mM peptide, 1 μl chymotrypsin (1 mg/ml, Sigma), and 0.5 μM purified protein. The absorbance was measured every 20 seconds for 10 min at 410 nm in a Genesys 10S UV-Vis spectrophotometer (Thermo) at 4°C.

### iHEK_P301L_ and sarkosyl-insoluble analysis

iHEK cells expressing tau P301L [24] for 9 days were transfected PCMV6-CyP40 (wild-type and H141E mutants) plasmids using Lipofectamine 2000 (Invitrogen). Forty-eight hours after transfection, the sarkosyl-insoluble fraction of tau was prepared as previously described [29]. The details are given in S1 Text.

### Mouse studies and tissue processing

For biochemical and tissue analyses, transgenic mice (rTg4510) overexpressing human tau P301L under a CaMKII promoter were injected with AAV9 vector at 6 months old (*N* = 8 [4 male, 4 female] for CyP40, *N* = 8 [4 male, 4 female] for GFP), and brains were harvested at 8 months old after perfusion using 0.9% saline. Hippocampi were dissected from the right hemisphere and snap frozen for biochemical processing. The left hemisphere was fixed in 4% paraformaldehyde overnight. Sucrose gradients up to 30% were used, and the tissue was sectioned with a sliding microtome to 25 μm thickness. All behavioral testing was performed at 5 months of age. For radial-arm water maze testing, rTg4510 along with wild-type control mice were injected with AAV9 at 3 months of age (*N* = 20 [10 wild type; 5 male, 5 female], [10 transgenic; 5 male, 5 female] for Cyp40, *N* = 18 [9 wild-type; 5 male, 4 female], [9 transgenic; 5 male, 4 female] for GFP). For fear conditioning analysis, rTg4510 along with wild-type control mice were injected with AAV9 at 3 months of age (*N* = 11 [6 wild type; 2 male, 4 female], [5 transgenic; 2 male, 3 female] for Cyp40, *N* = 11 [6 wild-type; 2 male, 4 female], [6 transgenic; 2 male, 4 female] for GFP). The experimenters were blind to both genotype and treatment of mice for biochemical, tissue, and behavioral analyses.

### Viral injection

Using stereotaxic equipment, virus was injected bilaterally into the mouse hippocampi at X = ±3.6, Y = −3.5, and Z = +2.68. Each injection delivered 2 μL of 1 x 10^12^ particles/mL of AAV9-GFP or AAV9-CyP40.

### Radial-arm water maze

A circular black tank with a 6-arm metal insert was filled with water, and a platform was submerged 1 cm below the surface of the water at the end of a designated goal arm. Animals were permitted 60 seconds to locate the platform, during which time an observer blind to treatment manually scored the number of errors. An error was defined as an entry into an incorrect arm or the absence of an arm choice within 15 seconds. Mice were trained over 2 days with 15 trials per day, which were divided into 5 sessions of 3 trials each.

### Fear conditioning

Two mild foot shocks (0.5 milliamps) were paired with an auditory conditioned stimulus (CS, white noise, 70 decibels) within a novel environment. The CS was given for 30 seconds before each foot shock (2 seconds). Twenty-four hours later, mice were placed into a novel context for 3 minutes without CS and then exposed to the CS for 3 minutes (cued).

### Immunohistochemistry and immunofluorescence

Tissue staining, imaging, and quantification are described in detail in S1 Text.

### Stereology

Neurons were stained with anti-NeuN and cresyl violet, and those positive for both were counted in the CA1 of the hippocampus. A computerized stereological system, connected to a Leica DM4000B microscope with a Prior motorized stage, was used to outline the area using distinct landmarks in the brain at 4× magnification [24,45]. Neurons were counted in this region by using randomly designated areas in the computer generated grid using a 100x oil immersion lens. Neurons were counted when they were located within the three-dimensional dissectors or touching the inclusion lines, and the top 1 μm and bottom 1 μm of tissue were excluded.

### Intrinsic disorder analysis

The intrinsic disorder predisposition of human CyP40 (UniProt ID: Q08752) was evaluated by 4 algorithms from the Predictor of Naturally Disordered Regions (PONDR) family: PONDR FIT [46], PONDR VLXT [47], PONDR VSL2 [48], and PONDR VL3 [49], as well as by the IUPred web server with its 2 versions for predicting long and short disordered regions [50]. The consensus disorder propensity of human CyP40 was evaluated by averaging disorder profiles of individual predictors.

### Statistical analysis

Analyses were performed using GraphPad Prism version 5.02. Group differences were analyzed with Student *t* test or one-way or two-way ANOVA as indicated in the figure legends. *P*-values less than 0.05, 0.01, or 0.001 are marked by 1, 2, or 3 asterisks (*), respectively. Error bars represent standard error of the mean (SEM). Data were examined for normal distribution and variance to determine if any datasets needed further analysis.

Sample sizes were not predetermined by statistical methods. Sample size estimates for animal studies were chosen based on previous experience with the rTg4510 mouse model. Data met assumptions for each test as analyzed by Shapiro-Wilk normality tests. Variation within groups was not significantly different as analyzed by an F-test of the equality of variances. Animals were randomized into experimental groups by an investigator blinded to genotype and treatment. Researchers were blinded for tissue analysis, stereology, and mouse behavior.

## Supporting information

S1 Fig Ratio-dependent CyP40 tau disaggregation. Incubations of increasing molar ratios of CyP40 to tau fibrils, as indicated, were monitored by Thioflavin T fluorescence. Samples were run in triplicate (n = 3). The numerical data used in figures can be found in S1 Data.
(TIF)

S2 Fig Inactivation of recombinant CyP40 with cyclosporin A (CsA) after disaggregation of tau fibrils. Cyclosporin A (dashed line) or DMSO (solid line) was administered to tau fibrils in the presence (red) or absence (black/grey) of CyP40 at 16 hours. Samples were run in duplicate (n = 2). The numerical data used in figure can be found in S1 Data.
(TIF)

S3 Fig Chymotrypsin-coupled PPIase activity assay. Curves represent No Enzyme (black), CyP40 (red), FKBP51 (blue), FKBP52 (purple) incubated with chymotrypsin (6mg/mL, pH 8.0) and substrate (Suc-AAPF-pNA, 100uM) over 300s. (One-way ANOVA, p < 0.0001, n = 2 independent preparations). The numerical data used in figure can be found in S1 Data.
(TIF)

S4 Fig Nanoparticle tracking analysis of CyP40. Nanoparticle tracking analysis assay of buffer (green), 7.5uM CyP40 (red), and 15uM CyP40 (orange).
(TIF)

S5 Fig CyP40 did not reduce cell viability. iHekP301L_P301L_ cell viability was monitored using an AlamarBlue assay following CyP40 (red) or vector (black) transfection ± tau induction by tetracycline. Results are expressed relative to vector without tau induction. Samples were run in triplicate (n = 3). The numerical data used in figure can be found in S1 Data.
(TIF)

S6 Fig AAV9 hippocampal injections and expression. (A) AAV9-GFP (green) or AAV9-CyP40 (red) injection locations within the hippocampus are indicated. Hippocampal regions are denoted, CA1, CA2, CA3, and Dentate Gyrus (DG). (B) A representative images of AAV9-GFP and CyP40 expression in hippocampi 2 months post-injection (scale bar 200 μm).
(TIF)

S7 Fig Expression levels of CyP40 protein from individual mouse brain lysates. Western blot analysis of CyP40 expression in AAV9-GFP and AAV9-CyP40 injected mice are compared to a standard curve generated with recombinant CyP40 protein (ng quantities indicated). Each lane represents an individual mouse. Western blot probed with anti-CyP40 antibody.
(TIF)

S8 Fig Nanoparticle analysis of α-synuclein and Aβ42 fibrils after CyP40 co-incubation. (A) Nanoparticle tracking analysis size distribution of A53T α-synuclein fibrils ± CyP40. (B) Representative images of (A). (C) Nanoparticle tracking analysis size distribution of Aβ42 fibrils ± CyP40. (D) Representative images of particles of (C).
(TIF)

S9 Fig PPIase activity assay and circular dichroism of wt and mut CyP40. (**A**) Coupled chymotrypsin assay of isomerase activity of wt CyP40 (red), mut CyP40 (teal), and No Enzyme (black) (n = 2 independent preparations). (**B**) Circular dichroism of wt CyP40 (red) and mut CyP40 (teal). The numerical data used in figure can be found in S1 Data.
(TIF)

S10 Fig Comparison of intrinsic disorder propensity of human and bovine CyP40 proteins. (A) Evaluating intrinsic disorder propensity of human CyP40 (UniProt ID: Q08752) by a series of per-residue disorder predictors. (B) Evaluating intrinsic disorder propensity of bovine CyP40 (UniProt ID: P26882). In these plots, disorder profiles generated by PONDR^®^ VLXT, PONDR^®^ VL3, PONDR^®^ VSL2, IUPred_short, IUPred_long, and PONDR^®^ FIT are shown by black, red, green, yellow, blue, and pink lines, respectively. Light pink shadow around the PONDR^®^ FIT shows error distribution. (C) Comparison of the mean disorder propensity of human black solid curve) and bovine CyP40 (red dashed curve). In plots A, B, and C, cyan shaded area shows position of the region with chaperone activity. Positions of three TPR motifs are shown as gray shaded areas. In these disorder analyses, the predicted intrinsic disorder scores above 0.5 are considered to correspond to the disordered residues/regions. (D) Pairwise sequence alignment of human and bovine CyP40 proteins (UniProt IDs Q08752 and P26882, respectively). Identical residues are indicated by star symbol, whereas colon and period symbols show similar residues. Sequences are colored according to the major physic-chemical properties of their residues, with red and green symbols corresponding to hydrophobic and polar residues, respectively, and with positively and negatively charged residues shown by pink and blue symbols, respectively.
(TIF)

S1 Text Supplemental experimental procedures. (DOCX)

S1 Data Underlying data. (XLSX)

## Abbreviations

α-syn: α-synuclein
Aβ42: amyloid_1-42_ peptide
AAV9: adeno-associated virus serotype 9
AD: Alzheimer’s disease
CS: conditioned stimulus
CsA: cyclosporin A
CyP: cyclophilin
CyP40: cyclophilin 40
FKBP: FK-506 binding protein
GAPDH: glyceraldehyde 3-phosphate dehydrogenase
GFP: green fluorescent protein
Hsp: heat-shock protein
HSQC: heteronuclear single quantum coherence
iHEK: inducible human embryonic kidney
MAPT: microtubule associating protein tau
mut: mutant
NMR: nuclear magnetic resonance
NTA: nanoparticle tracking analysis
PD: Parkinson’s disease
PDB: Protein Data Bank
PONDR: Predictor of Naturally Disordered Regions
PPIase: peptidyl-prolyl isomerase
RFU: relative fluorescence unit
RMSD: root-mean-square deviation
SEM: standard error of the mean
TEM: transmission electron microscopy
TPR: tetracopeptide repeat
ThT: Thioflavin T
wt: wild type
