# Supplementary figures and images for: Human cyclophilin 40 unravels neurotoxic amyloids
Source: PLoS Biol. 2017 Jun 27;15(6):e2001336. doi: 10.1371/journal.pbio.2001336 (PMC5486962; doi:10.1371/journal.pbio.2001336)

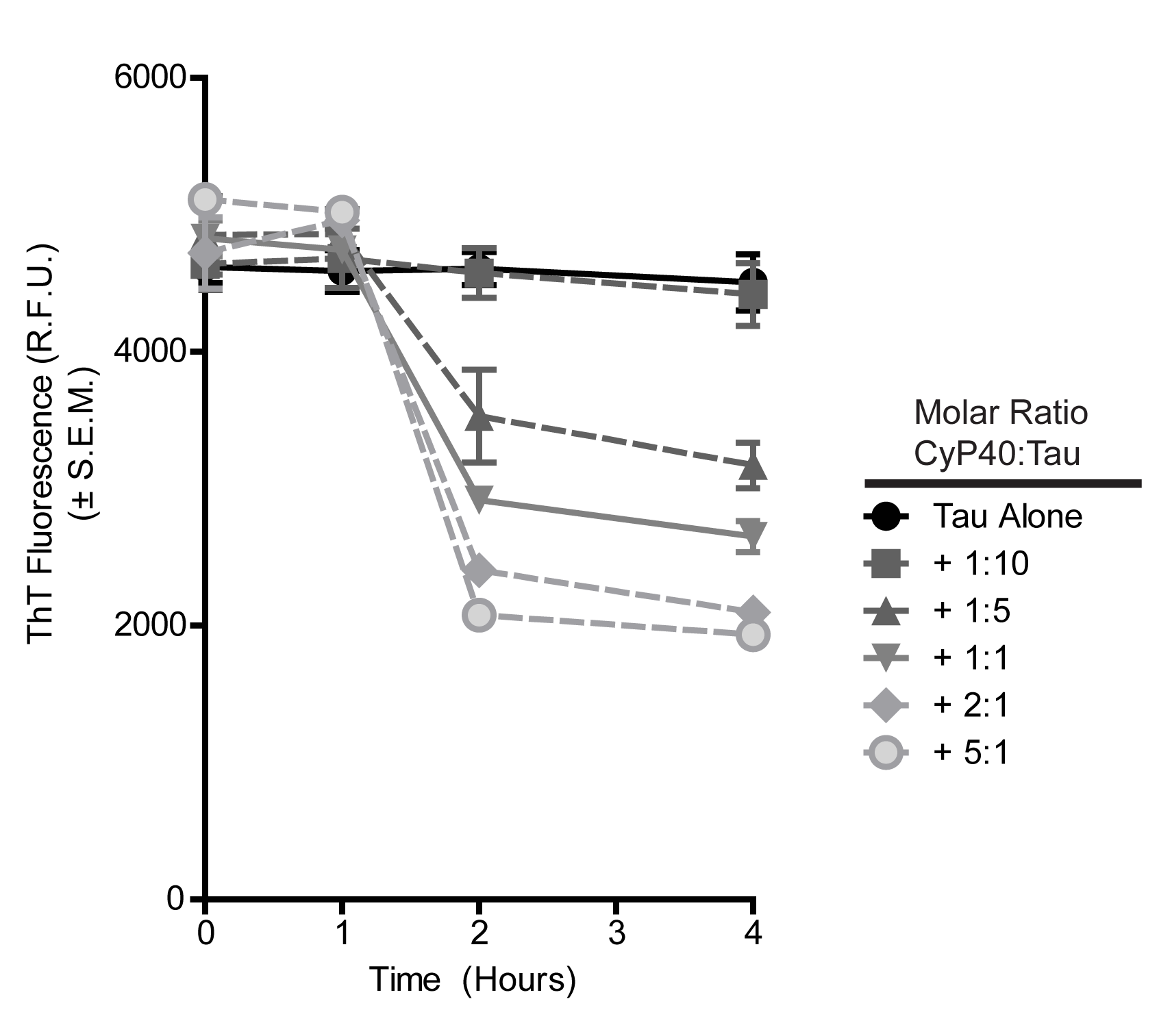

Supplement: S1 Fig — Incubations of increasing molar ratios of CyP40 to tau fibrils, as indicated, were monitored by Thioflavin T fluorescence. Samples were run in triplicate (n = 3). The numerical data used in figures can be found in S1 Data. (TIF) [file pbio.2001336.s001.tif]

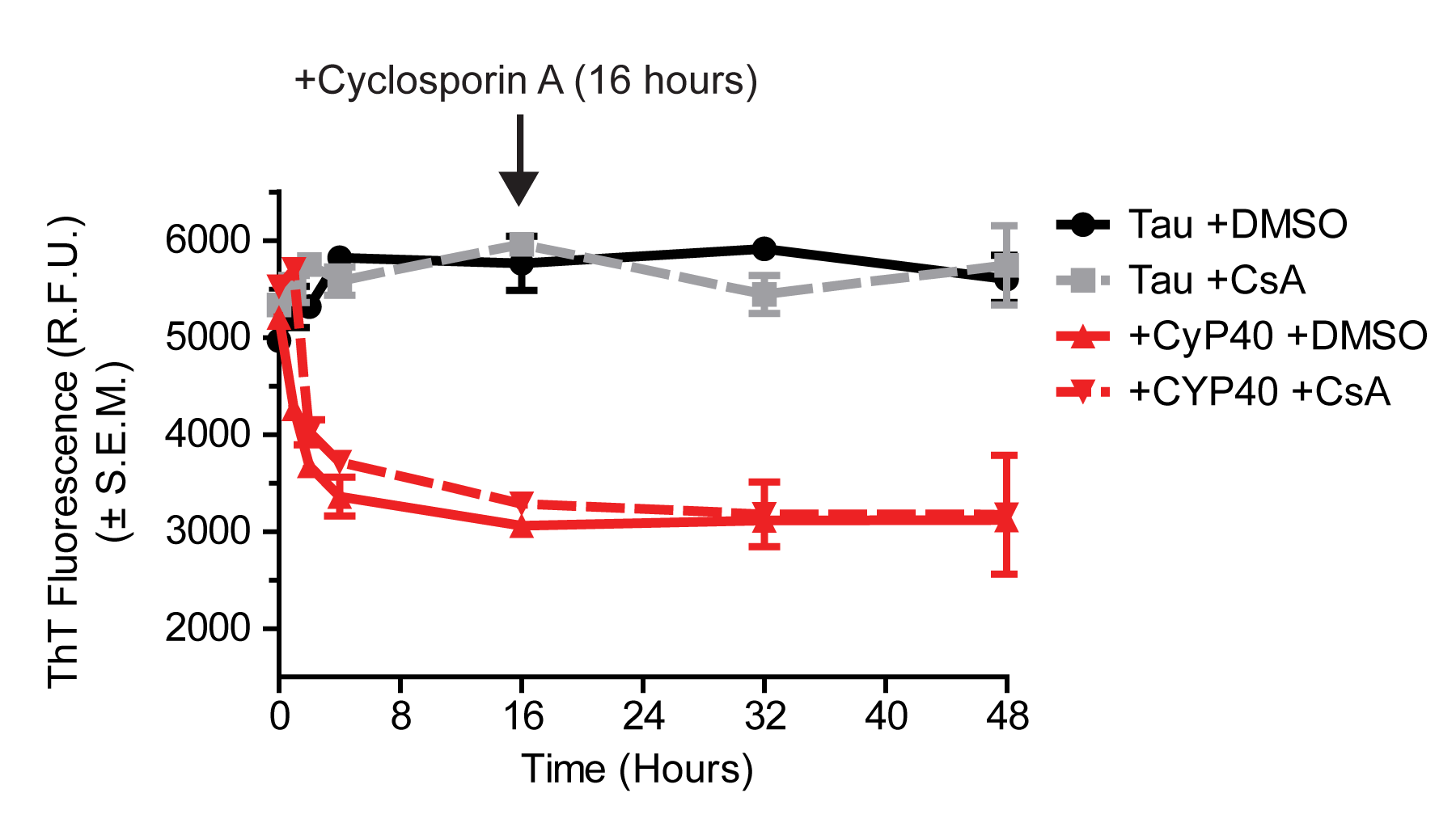

Supplement: S2 Fig — Cyclosporin A (dashed line) or DMSO (solid line) was administered to tau fibrils in the presence (red) or absence (black/grey) of CyP40 at 16 hours. Samples were run in duplicate (n = 2). The numerical data used in figure can be found in S1 Data. (TIF) [file pbio.2001336.s002.tif]

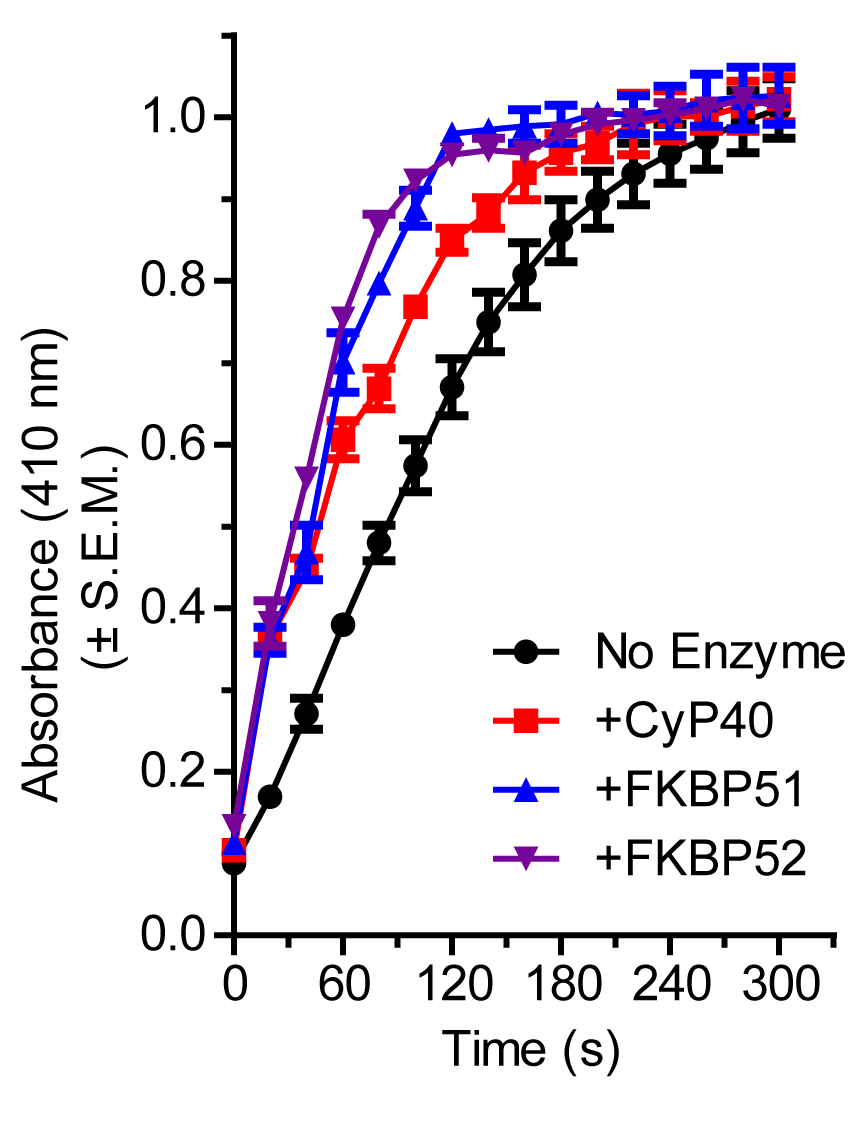

Supplement: S3 Fig — Curves represent No Enzyme (black), CyP40 (red), FKBP51 (blue), FKBP52 (purple) incubated with chymotrypsin (6mg/mL, pH 8.0) and substrate (Suc-AAPF-pNA, 100uM) over 300s. (One-way ANOVA, p < 0.0001, n = 2 independent preparations). The numerical data used in figure can be found in S1 Data. (TIF) [file pbio.2001336.s003.tif]

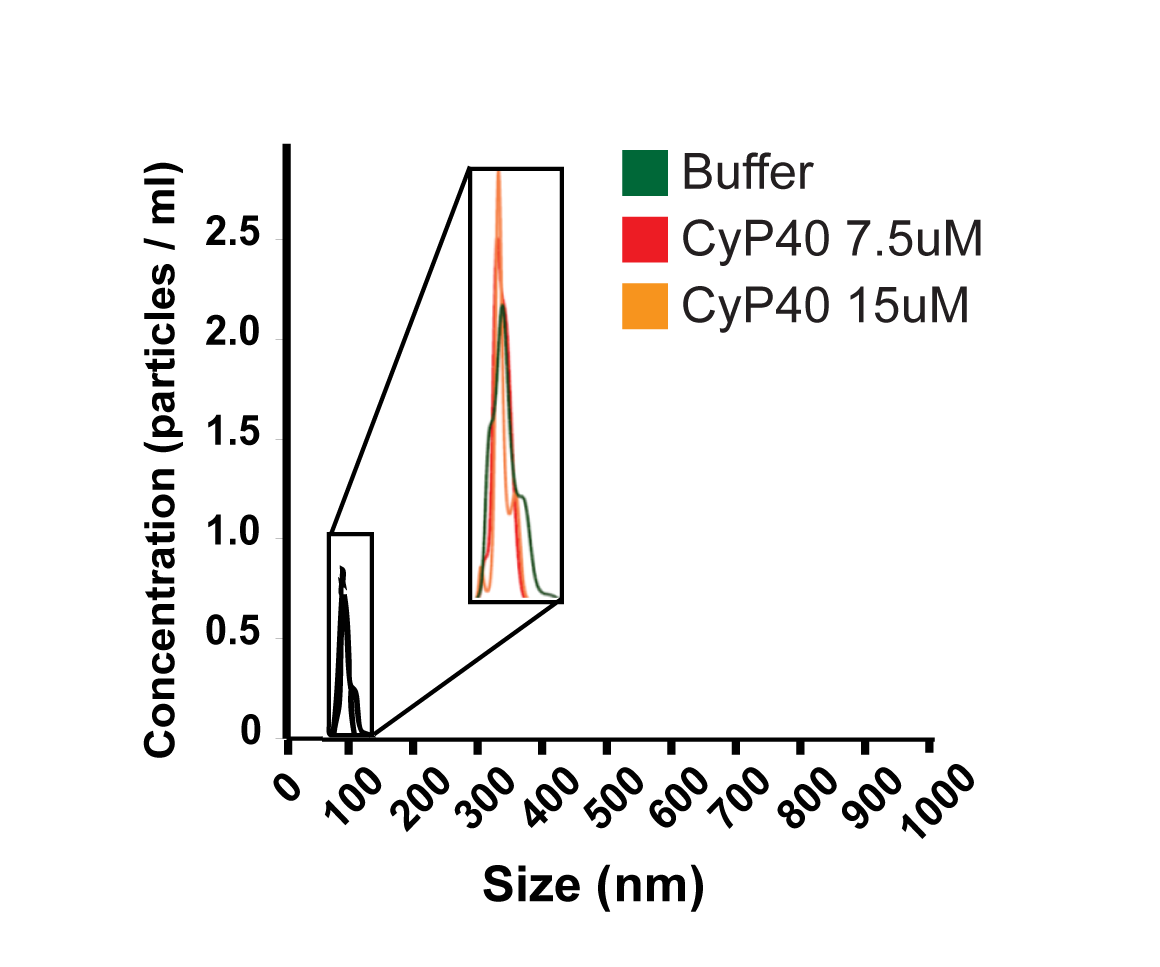

Supplement: S4 Fig — Nanoparticle tracking analysis assay of buffer (green), 7.5uM CyP40 (red), and 15uM CyP40 (orange). (TIF) [file pbio.2001336.s004.tif]

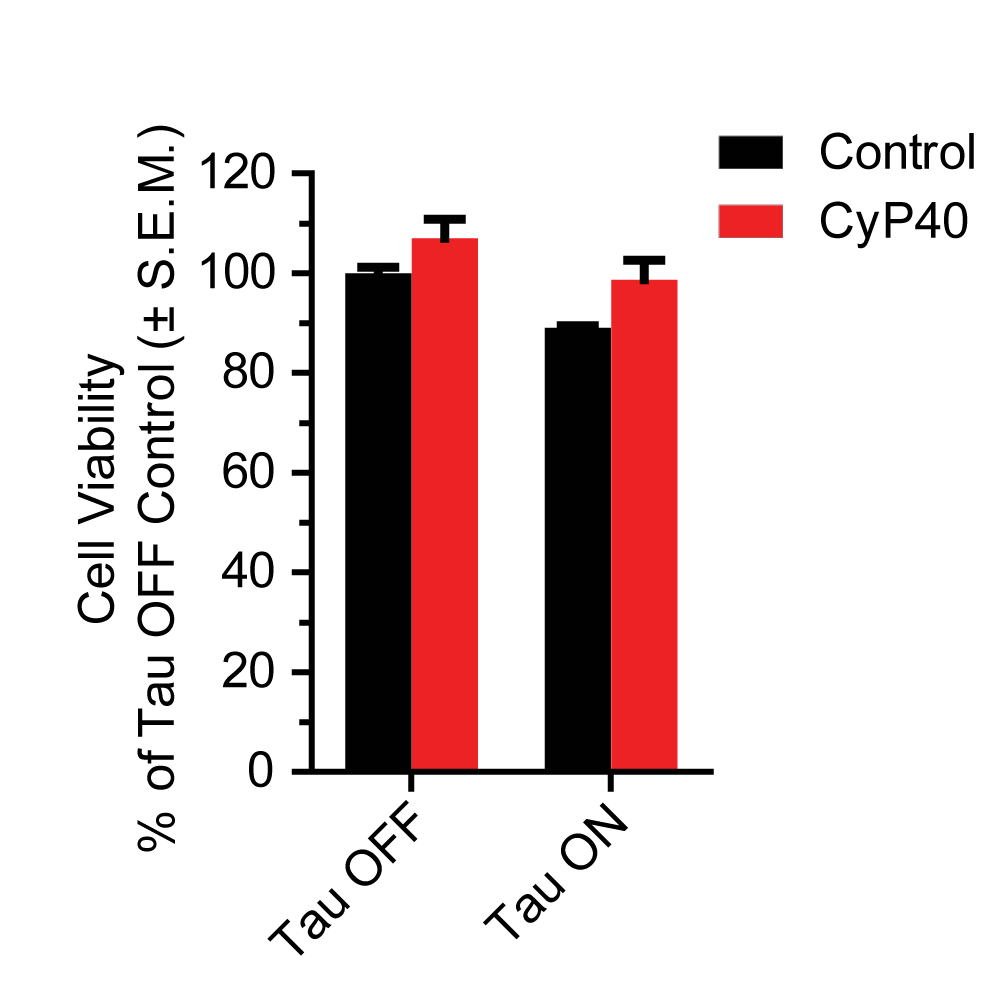

Supplement: S5 Fig — iHekP301LP301L cell viability was monitored using an AlamarBlue assay following CyP40 (red) or vector (black) transfection ± tau induction by tetracycline. Results are expressed relative to vector without tau induction. Samples were run in triplicate (n = 3). The numerical data used in figure can be found in S1 Data. (TIF) [file pbio.2001336.s005.tif]

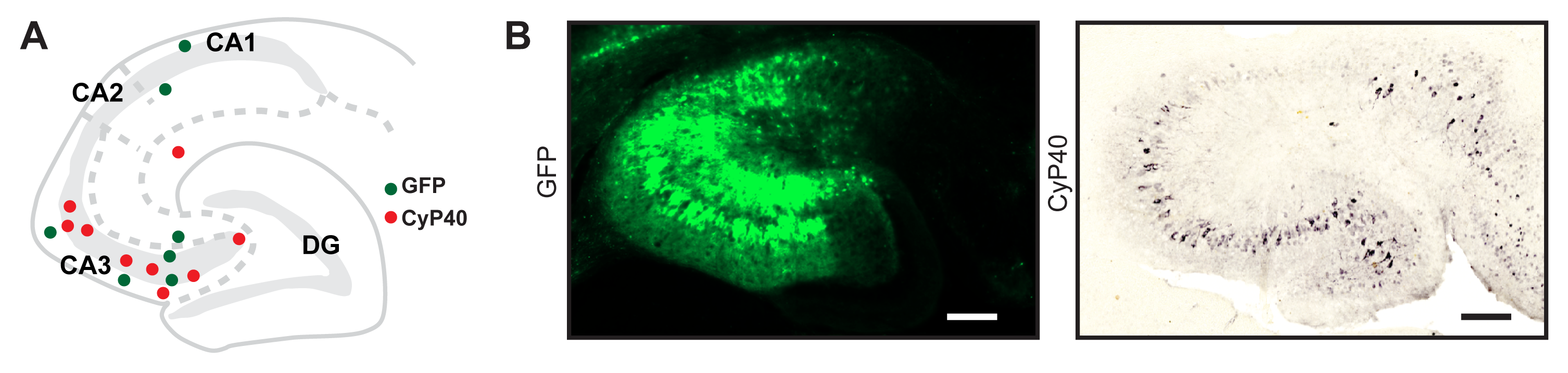

Supplement: S6 Fig — (A) AAV9-GFP (green) or AAV9-CyP40 (red) injection locations within the hippocampus are indicated. Hippocampal regions are denoted, CA1, CA2, CA3, and Dentate Gyrus (DG). (B) A representative images of AAV9-GFP and CyP40 expression in hippocampi 2 months post-injection (scale bar 200 μm). (TIF) [file pbio.2001336.s006.tif]

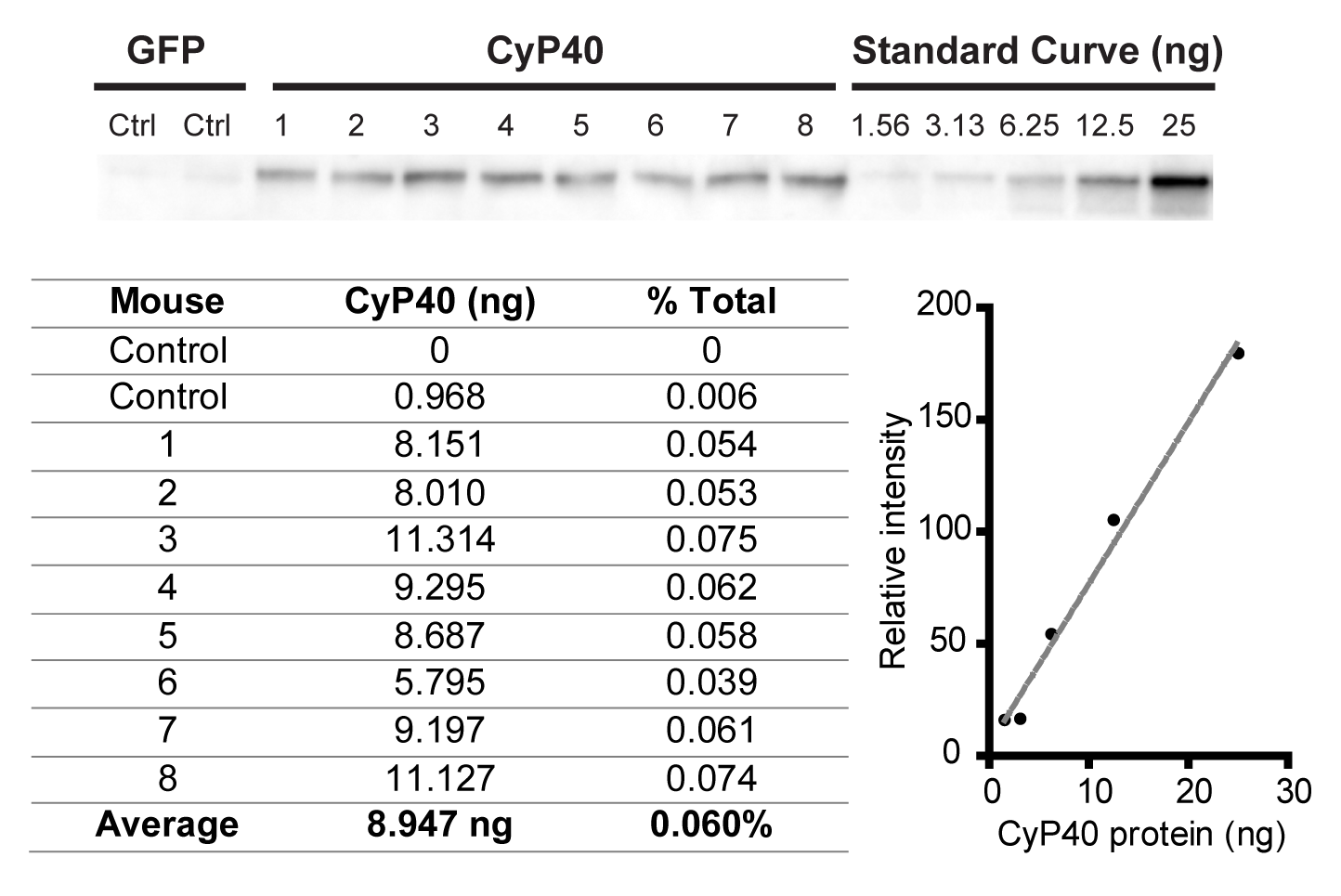

Supplement: S7 Fig — Western blot analysis of CyP40 expression in AAV9-GFP and AAV9-CyP40 injected mice are compared to a standard curve generated with recombinant CyP40 protein (ng quantities indicated). Each lane represents an individual mouse. Western blot probed with anti-CyP40 antibody. (TIF) [file pbio.2001336.s007.tif]

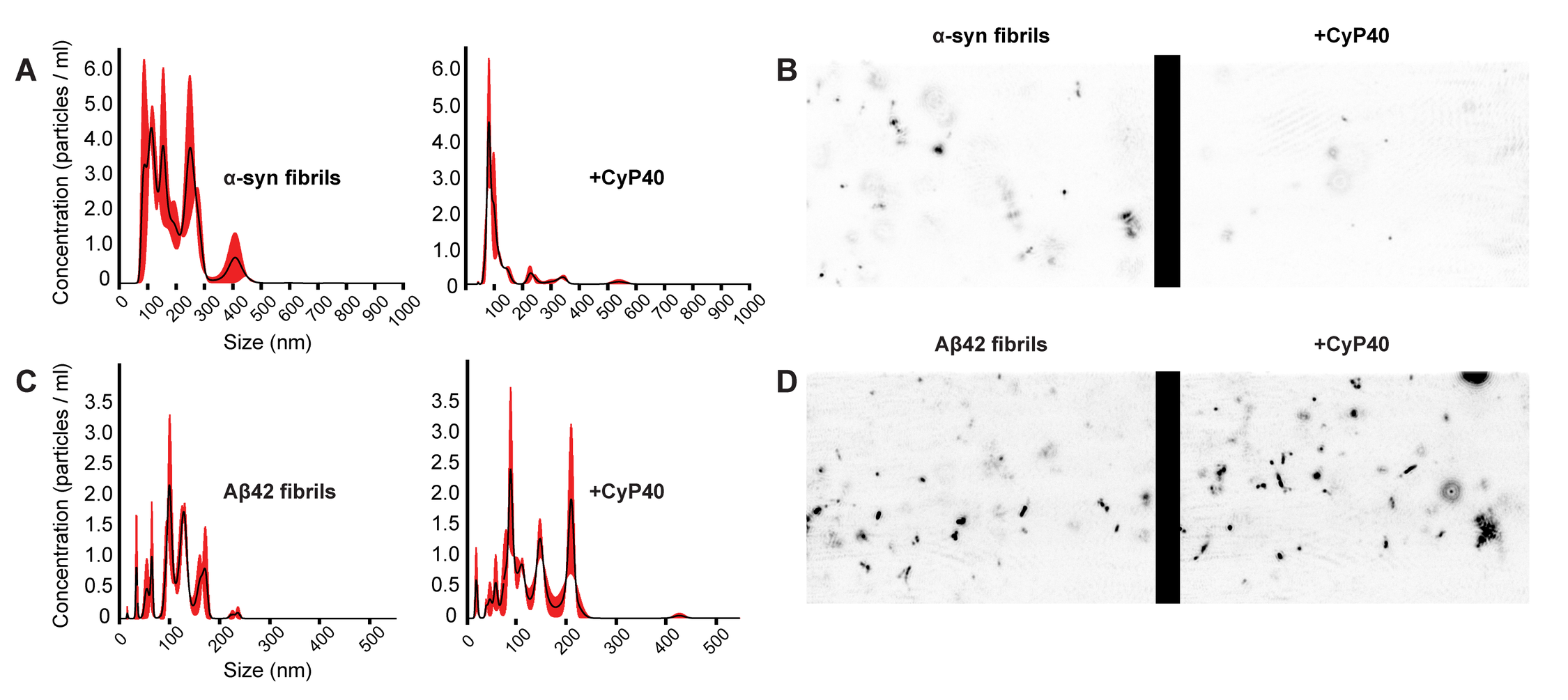

Supplement: S8 Fig — (A) Nanoparticle tracking analysis size distribution of A53T α-synuclein fibrils ± CyP40. (B) Representative images of (A). (C) Nanoparticle tracking analysis size distribution of Aβ42 fibrils ± CyP40. (D) Representative images of particles of (C). (TIF) [file pbio.2001336.s008.tif]

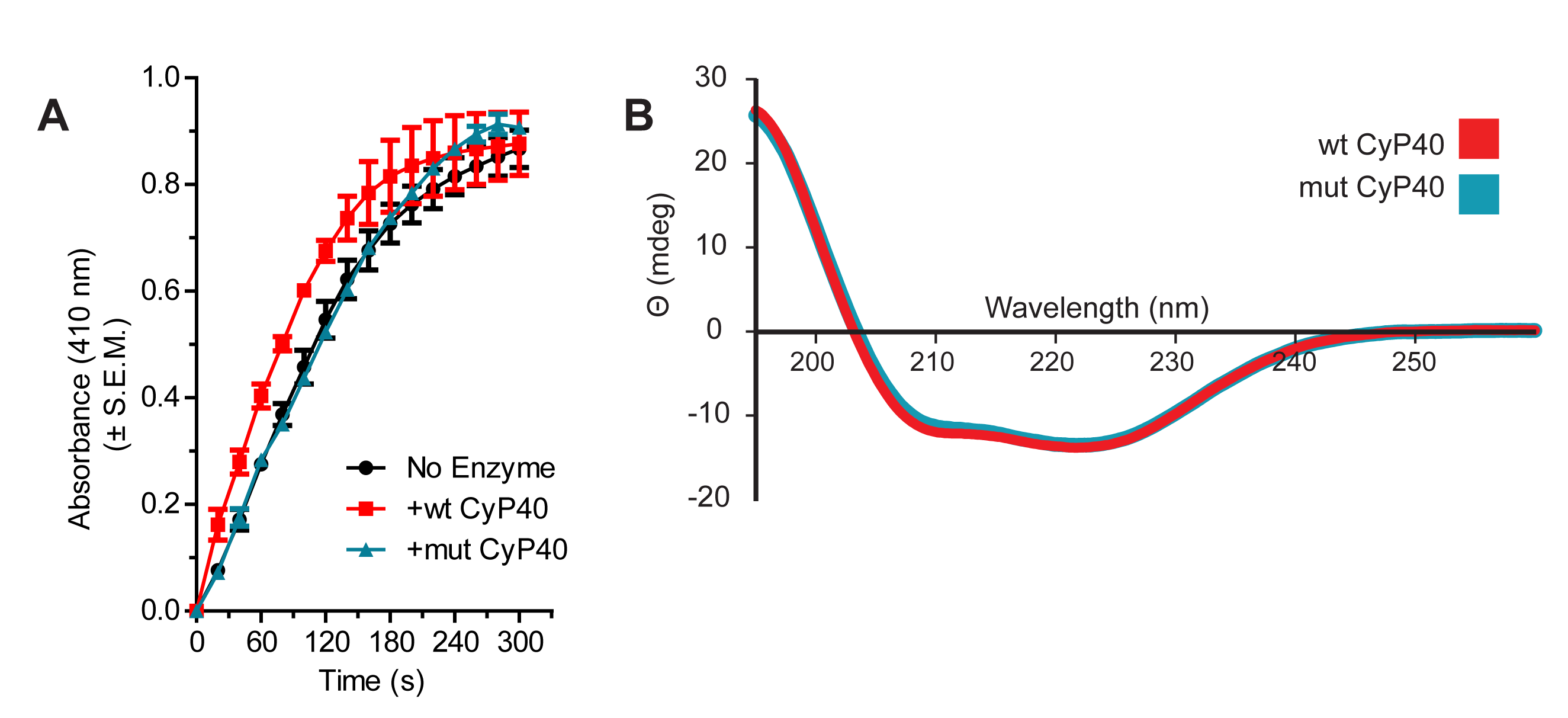

Supplement: S9 Fig — (A) Coupled chymotrypsin assay of isomerase activity of wt CyP40 (red), mut CyP40 (teal), and No Enzyme (black) (n = 2 independent preparations). (B) Circular dichroism of wt CyP40 (red) and mut CyP40 (teal). The numerical data used in figure can be found in S1 Data. (TIF) [file pbio.2001336.s009.tif]

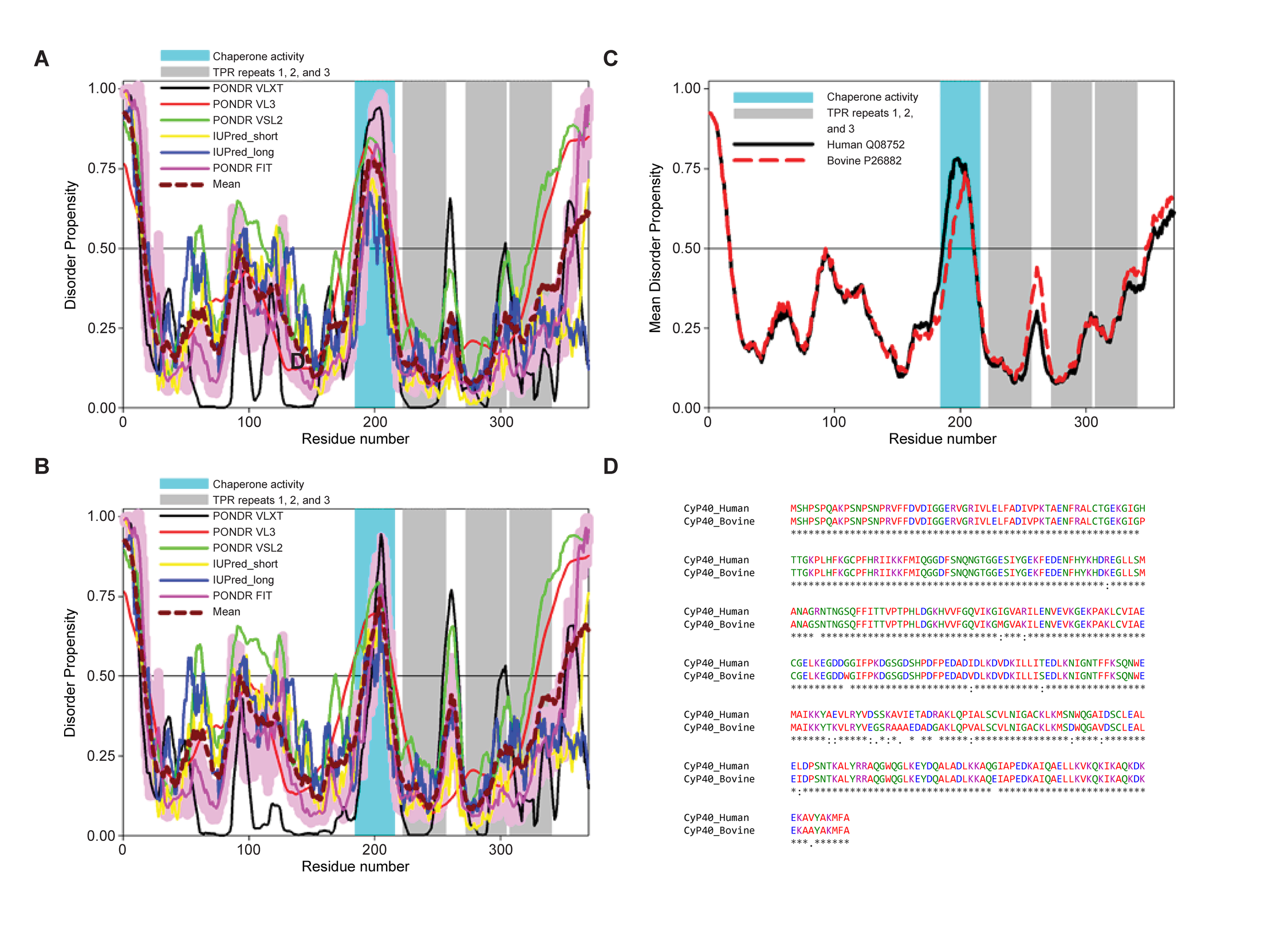

Supplement: S10 Fig — (A) Evaluating intrinsic disorder propensity of human CyP40 (UniProt ID: Q08752) by a series of per-residue disorder predictors. (B) Evaluating intrinsic disorder propensity of bovine CyP40 (UniProt ID: P26882). In these plots, disorder profiles generated by PONDR® VLXT, PONDR® VL3, PONDR® VSL2, IUPred_short, IUPred_long, and PONDR® FIT are shown by black, red, green, yellow, blue, and pink lines, respectively. Light pink shadow around the PONDR® FIT shows error distribution. (C) Comparison of the mean disorder propensity of human black solid curve) and bovine CyP40 (red dashed curve). In plots A, B, and C, cyan shaded area shows position of the region with chaperone activity. Positions of three TPR motifs are shown as gray shaded areas. In these disorder analyses, the predicted intrinsic disorder scores above 0.5 are considered to correspond to the disordered residues/regions. (D) Pairwise sequence alignment of human and bovine CyP40 proteins (UniProt IDs Q08752 and P26882, respectively). Identical residues are indicated by star symbol, whereas colon and period symbols show similar residues. Sequences are colored according to the major physic-chemical properties of their residues, with red and green symbols corresponding to hydrophobic and polar residues, respectively, and with positively and negatively charged residues shown by pink and blue symbols, respectively. (TIF) [file pbio.2001336.s010.tif]
