# Supplementary material for: Human cyclophilin 40 unravels neurotoxic amyloids
Source: PLoS Biol. 2017 Jun 27;15(6):e2001336. doi: 10.1371/journal.pbio.2001336 (PMC5486962; doi:10.1371/journal.pbio.2001336)
Supplement: S1 Text — (DOCX) [file pbio.2001336.s011.docx]

**S1 Text**

**Supplemental Experimental Procedures**

**Recombinant protein purification**

Recombinant human tau P301L, CyP40, CyP40 H141E, FKBP51, and FKBP52 were cloned into PET28A plasmids with a TEV protease sequence, and then transformed into competent *E.coli* BL21 cells and plated on kanamycin-agar plates and incubated at 37°C overnight (~16 hrs). A single colony was used to inoculate a 10 mL starter culture of LB broth supplemented with kanamycin. After 8-12 hrs, starter cultures were used to inoculate 1 L of LB-kanamycin broth and cultures were grown to an OD600 of 0.8 (~3 hrs). At this point, cultures were induced with 1 mM (final concentration) IPTG and grown a further 3 hrs. Pellets were spun down at 3500 x *g* for 30 min and supernatant was discarded by aspiration. Pellets were then resuspended in Nickel chromatography running buffer (20 mM Tris-HCl pH 8.0, 500 mM NaCl, 10 mM Imidazole) and frozen for up to 3 months at -80°C.

Pellets were then thawed on ice and subsequently lysed by sonication and spun at 50,000 x *g* for 1 hr. After filtration of the supernatant with a 0.02 µm filter, a standard gravity nickel column using Ni-NTA resin (Fisher #PI88222) was performed. TEV protease was then added to the elution fraction, placed into a 3,000 MW cutoff dialysis bag and into TEV buffer overnight at 4°C. The solution was then dialyzed back into nickel chromatography running buffer and a 2^nd^ nickel purification column was ran. We then performed size exclusion chromatography using a HiLoad 16/600 Superdex 200pg column and fractions were pooled and concentrated. Proteins were then dialyzed into appropriate buffer (PBS or Sodium Acetate). Proteins were estimated to be >95% pure by coomassie staining.

**iHEK_P301L_ and sarkosyl-inoluble analysis**

The tetracycline-inducible HEK293 cell line (iHEK) was generated by the insertion of human tau P301L 4R0N DNA into a pCDNA 4/TO plasmid vector (Invitrogen). The tau/TO plasmid construct was transfected into the T-REx HEK cell line (Invitrogen) which stably expresses the tetracycline repressor protein. After zeocin selection (200 µg/ml), the colonies were picked up and the expression of Tau was detected by Western blot with and without tetracycline (1 µg/ml). Cell lines were found to be free of mycoplasma contamination.

To investigate the effect of CyP40 on the level of aggregated, insoluble tau, tau expression was induced for 9 days and then transfected with PCMV6-CyP40 (wild-type and H141E mutants) plasmids using Lipofectamine 2000 (Invitrogen) following the manufacturer’s instructions. 48 h after transfection, the sarkosyl-insoluble fraction of Tau was prepared as previously described [29].

**Immunohistochemistry and immunofluorescence**

Tissue was stained free floating. Tissue sections were incubated in PBS supplemented with 10% MeOH and 3% H_2_O_2_ to block endogenous peroxidases. Following PBS washes, tissue was permeabilized by 0.2% Triton-X-100 with 1.83% lysine and 4% serum in PBS for 30 min. Tissue was then incubated at room temperature overnight in primary antibody. The following primary antibodies were used: anti-CyP40 (1:2000, Pierce PA3-023), T22 (1:700, Dr. Rakez Kayed), and anti-H150 tau (1:30000, Santa Cruz SC-5587). Following three PBS washes, biotinylated goat anti-rabbit (Southern Biotech) secondary (1:10000) was added for 2 hrs. Prior to peroxidase development, an ABC kit (Vectastain) was used to increase visibility. Following PBS washes, tissue was incubated with 0.05% diaminobenzidine plus 0.5% nickel and developed with 0.03% H_2_O_2_. Tissue sections were then mounted and allowed to dry overnight before dehydration in alcohol gradients. Slides were cleared by Histoclear then coverslipped with DPX (distyrene, plasticizer, and xylene) mountant as a synthetic resin mounting media.

Sections stained for stereology were blocked and permeabilized as described above and incubated overnight with biotinylated NeuN (1:3000, EMD Millipore MAB377B) at room temperature. Following PBS washes, ABC conjugation, and peroxidase development tissue was mounted on glass slides and dried overnight. These sections were then counter-stained with cresyl violet (nissl) by incubating with 0.05% cresyl violet briefly and then quickly destaining with 0.3% acetic acid in water prior to dehydration.

For Gallyas silver staining, tissue slices were mounted on glass slides and dried overnight. These slides were then incubated in a 0.003% potassium permanganate solution for 10 min. After rinsing with water the sections were incubated from 1-2 min in a 2.0% oxalic acid solution then rinsed thoroughly in water. Slides were then incubated in a 5.0% sodium metaperiodate solution for 5 min and again rinsed in water. Slides were then treated with an alkaline silver iodide solution (1 M sodium hydroxide, 0.6 M potassium iodide, 0.053% silver nitrate) for 1 min, then rinsed three times with a 0.5% acetic acid solution. Staining was developed by combining solutions A (5% sodium carbonate), B (0.024 M ammonium nitrate, 0.012 M silver nitrate, 0.003 M tungstosilicic acid), and C (0.024 M ammonium nitrate, 0.012 M silver nitrate, 0.003 M tungstosilicic acid, 0.25% formaldehyde) in a 2:1:1 ratio, adding B and C to solution A and incubating for 10-30 min. The slides were then rinsed three times in 0.5% acetic acid, then water. The slides were then incubated in gold tone for 3-4 min and again rinsed in water, then a 1% sodium thiosulphate solution for 5 min and a final rinse in water before they were dehydrated and coverslipped using DPX.

**Tissue imaging and quantification**

An Axio Scan.Z1 (Zeiss) slide scanning microscope was used to image all tissue. Bright field analysis was performed using Zeiss Neuroquant IAE analysis software. This program was used to outline regions of interest from the entire slide. Then, thresholds were set manually until only positive cells, as determined by the analyzer, were selected with as little non-specific areas selected as possible. Using the batch process option, the Area Ratio of positive cells within the regions of interest was automatically calculated for each stained group.
